# A multi-omics features-based approach integrating immunogenicity and inflammation enhances immunotherapy benefit in clear cell renal cell carcinoma

**DOI:** 10.3389/fcell.2025.1596719

**Published:** 2026-01-20

**Authors:** Yanfeng Xue, Feng Han, Shuqing Wei, Yiqun Zhang, Nan Wang, Ling Liu, Zhen Chen, Zhihua Pei, Hailong Hao

**Affiliations:** 1 Department of Special Needs Medicine, Cancer Hospital Affiliated to Shanxi Medical University/Shanxi Province Cancer Hospital/Shanxi Hospital Affiliated to Cancer Hospital, Chinese Academy of Medical Sciences, Taiyuan, China; 2 Department of Information Management, Cancer Hospital Affiliated to Shanxi Medical University/Shanxi Province Cancer Hospital/Shanxi Hospital Affiliated to Cancer Hospital, Chinese Academy of Medical Sciences, Taiyuan, China; 3 Department of Comprehensive Medicine, Cancer Hospital Affiliated to Shanxi Medical University/Shanxi Province Cancer Hospital/Shanxi Hospital Affiliated to Cancer Hospital, Chinese Academy of Medical Sciences, Taiyuan, China; 4 Frontier Science Center for Stem Cell Research, School of Life Sciences and Technology, Tongji University, Shanghai, China; 5 School of Medicine, Xiamen University, Xiamen, China; 6 College of Bioinformatics Science and Technology, Harbin Medical University, Harbin, Heilongjiang, China; 7 Department of Rheumatology and Immunology, The Second Affiliated Hospital of Fujian Medical University, Quanzhou, China; 8 Hubei Key Laboratory of Agricultural Bioinformatics, College of Informatics, Huazhong Agricultural University, Wuhan, China; 9 Department of Urology, Cancer Hospital Affiliated to Shanxi Medical University/Shanxi Province Cancer Hospital/Shanxi Hospital Affiliated to Cancer Hospital, Chinese Academy of Medical Sciences, Taiyuan, China

**Keywords:** clear cell renal cellcarcinoma(ccRCC), immune checkpoint blockade (ICB), immunotherapy response, machine learning, multi-omics

## Abstract

**Background:**

Programmed cell death 1 (PD-1) or PD-ligand 1 (PD-L1) blocker-based strategies have improved the survival outcomes of clear cell renal cell carcinomas (ccRCCs) in recent years, but only a small number of patients have benefited from them.

**Methods:**

In this study, we developed a multi-omics machine learning model based on inflammatory and immune signatures (TIs) to predict the response and survival of ccRCC patients to immune checkpoint blockade (ICB) therapy. The research collected RNA-seq and single-cell RNA-seq (scRNA-seq) data from more than 1,900 patients with autoimmune nephropathy and analyzed the genomic and transcriptome profiles of ccRCC patients. The predictive power of the method was validated in more than 1,000 ccRCC patients treated with ICB, and compared to single biomarkers (e.g., PD-L1 expression, TMB).

**Results:**

Inflammatory signaling was found to be strongly associated with ICB outcome, and 716 inflammation-related genes were identified that are enriched in the “lymphocyte activation regulation” pathway. The findings suggested that ccRCC patients can be categorized into two subtypes with different treatment responses and prognosis by these features. In addition, the TIs-ML model exhibited superior predictive capabilities compared to an individual biomarker (AUC > 0.997) across multiple independent datasets. It demonstrated the capacity to accurately differentiate between responders and non-responders. Furthermore, the model performed more effectively than existing genetic models and functional scores in predicting survival.

**Conclusion:**

We propose a TIs-ML prediction model based on multi-omics features that can effectively predict ICB treatment response in ccRCC patients. The model integrates inflammatory and immune features, and its high generalization ability was validated in multiple cohorts. Overall, the TIs-ML approach provides a novel method for guiding precise immunotherapy in ccRCC.

## 1 Introduction

Immune checkpoint blockades (ICBs) that block programmed death 1 (PD-1), programmed cell death ligand 1 (PD-L1), or cytotoxic T lymphocyte antigen 4 (CTLA4) have ushered in a new era in the treatment of advanced cancer (Maio et al., 2022; Shitara et al., 2022). Notably, PD-(L)1-based therapies have become the standard-of-care option for advanced clear cell renal cell carcinomas (ccRCCs), regardless of the number of prior lines of therapy received (Motzer et al., 2015; Motzer et al., 2019; Rini et al., 2019a; Choueiri et al., 2021; Lee et al., 2021). However, only a subset of ccRCC patients (20%–50%) respond to immune monotherapy or combination therapies (Motzer et al., 2015; Motzer et al., 2019; Rini et al., 2019b; Motzer et al., 2018). Therefore, a comprehensive approach for identifying biomarkers that are associated with potential immunotherapy responders or survival-benefit patients is a significant unmet medical need in ccRCC with ICB treatment.

The major challenge is to find comprehensive biomarkers that can predict response and survival to immunotherapy alone or in combination for ccRCC, regardless of lines of therapy. For example, PD-L1 expression, which belongs to proteomics, and tumor mutation burden (TMB), which is defined by genomic alterations, both are predictive biomarkers of response to PD-1/PD-L1 inhibitors in non-small cell lung cancer, melanoma, colorectal cancer, and head and neck cancers (Reck et al., 2016; Hodi et al., 2018; Hsu et al., 2017; Rizvi et al., 2015; Samstein et al., 2019; Marabelle et al., 2020; Meiri et al., 2020), but applying them to predict the efficacy of immunotherapy for ccRCC undergoing ICB therapy has been continuously questioned (Labriola et al., 2020; Motzer et al., 2020a). Bulk RNA sequencing (RNA-seq) or single-cell RNA sequencing (scRNA-seq)-based multi-gene prognostic models and comprehensive somatic alteration signatures through genomics analysis are based on one single omics, improving the accuracy of ICB response prediction to some extent (Labriola et al., 2020; Motzer et al., 2020a; McDermott et al., 2018; Krishna et al., 2021). Indeed, recent studies have demonstrated that immune infiltration interacts with genomic features in ccRCC (Braun et al., 2020; Motzer et al., 2020b). Multi-omics-based machine learning (ML) models outperform single-omics methods in predicting therapy efficacy, which has been proven in esophageal cancer and breast cancer (Sammut et al., 2022; Liu et al., 2023). These biomarkers identified in patients with ICB can only partially explain the response to immunotherapy treatment (Kong et al., 2022), indicating that a more integrated multi-omics biomarker is needed for ccRCC patients.

Nonetheless, the novel multi-omics features need to be investigated not only from immunogenic characteristics in oncological patients with ICB, but also from inflammatory profiles in non-oncological patients with autoimmune disease. The kidney has a more complex immune environment than other organs, which is vulnerable to autoimmune attacks (Lech and Anders, 2013; He et al., 2020). There is increasing evidence that the inflammatory signatures of autoimmune nephropathy are strongly correlated with the efficacy of immunotherapy for patients with advanced malignant tumors, especially in ccRCC (Grandi and Tramontano, 2018; Masetti et al., 2021; Khandwala et al., 2025). The potential reason is that immune-related adverse effects (irAEs) derived from immunotherapy, which can predict the prognosis of immunotherapy, are similar in pathogenesis and clinical therapy to autoimmune nephropathy (Puzanov et al., 2017; Haanen et al., 2017; Socinski et al., 2023; van Not et al., 2022; Vitzthum von Eckstaedt et al., 2024; Deng et al., 2025). However, there is a lack of relevant literature on integrating ML-based inflammatory and immunogenic multi-omics approaches to improve the predictive specificity and robustness of ccRCC with ICB regimens.

In this present research, we offer an inflammatory and immunogenic-based (TIs) multi-omics ML framework that can (i) generate robust predictions about response and survival on ICB datasets, (ii) construct predictions specifically for ccRCC with ICB, and (iii) develop overall survival predictions in ccRCC. Specifically, we identified three types of inflammatory biomarkers in more than 1900 patients with autoimmune nephropathy using RNA-seq and scRNA-seq data, and three types of immunogenic signatures by genomics analysis in ccRCC with immunotherapy. Ultimately, with the TIs-ML model, we can reliably distinguish responders or survival-benefit patients in over 1,000 ccRCCs with ICB. Predictive models based on individual 6-type features have presented a great variation in performance, while the ensemble model outperforms either individual classifier. The performance of the aggregate model was superior to the biomarkers identified in immunotherapy-treated patients, such as tumor mutation burden (TMB), PD-L1 expression, and tumor immune microenvironment (TIME). Moreover, we obtained 716 genes related to inflammation in autoimmune nephropathy, which were enriched in the largest pathway as “regulation of lymphocyte activation.” Using these genes, we clustered ccRCC into two categories, which have different treatment responses and prognosis preferences. As a result, we find that the inflammatory profiles of autoimmune nephropathy are strongly associated with the outcome of immunotherapy in ccRCC, and our integrated model improves the prediction of the ICB response and survival.

## 2 Methods

### 2.1 Data collection

We systematically searched the transcriptome data of five autoimmune nephropathy from 36 datasets in the Gene Expression Omnibus (GEO) database (Edgar et al., 2002) based on the review articles (Kant et al., 2022; Boesen and Kakalij, 2021). These datasets included bulk RNA datasets of 5 Lupus Nephritis (LN) datasets (GSE112943, GSE200306, GSE32591, GSE81622, GSE99967), 7 IgA Nephropathy (IgAN) datasets (GSE175759, GSE37460, GSE104954, GSE141295, GSE116626, GSE35489, GSE115857), 10 Membranous Nephropathy (MN) datasets (GSE216841, GSE200828, GSE200818, GSE182380, GSE197307, GSE133288, GSE115857, GSE108113, GSE104954, GSE104948), 8 Focal Segmental Glomerulosclerosis (FSGS) datasets (GSE200828, GSE200818, GSE182380, GSE197307, GSE133288, GSE108113, GSE104954, GSE104948) and 3 Anti-neutrophil Cytoplasmic Antibody-associated Vasculitis (ANCA-AAV) datasets (GSE108113, GSE104954, GSE104948), as well as three scRNA datasets of IgAN (GSE171314), MN (GSE171458), and LN [GSE121893 (Arazi et al., 2019)] (Supplementary Data S1). It should be noted that we excluded cases and controls that were less than five in bulk datasets and those with obviously non-active LN in GSE99967. We also excluded one IgAN patient with microproteinuria in GSE17131.

For the immune datasets, we collected transcriptome and genomic data with clinical annotations from three ccRCC cohorts: IMmotion151 (Buttner et al., 2022) (atezolizumab + bevacizumab versus Sunitinib; first-line) from the European Genome-phenome Archive (EGA) dataset, CheckMate (nivolumab versus everolimus; latter-line) from the Supplementary Materials provided by Braun et al. (2020), and JAVELIN (avelumab + axitinib versus sunitinib; first-line) from the Supplementary Materials provided by Motzer et al. (2020a). We also gathered four clinically annotated transcriptome immune cohorts of urothelial carcinoma (UC) [IMvigor210 (Balar et al., 2017), treated by atezolizumab, n = 208], NSCLC [POPLAR (Fehrenbacher et al., 2016), treated by atezolizumab, n = 81], RCC [IMmotion150 (McDermott et al., 2018), treated by atezolizumab alone or in combination with bevacizumab, n = 162], multi-cancer [PCD4989g (Herbst et al., 2014), treated by atezolizumab, n = 206]. Patients with clearly four treatment outcomes of “CR, PR, PD, SD” were enrolled except JEVELIN which with known survival time records were enrolled.

Mutation and clinical data of ccRCC from The Cancer Genome Atlas (TCGA) database were downloaded from cbioportal (https://www.cbioportal.org/study/summary?id=kirc_tcga). This study generates no new data, so no ethics approval is needed. The Supplementary Data S1 of this study contains comprehensive information on all of the data.

### 2.2 Bulk RNA-seq data process and analysis

All expression array data were log2-transformed using the GEO protocol. RNA-seq count data were normalized to FPKM for each gene based on the gencode v22 and log2-transformed, subsequently. The analysis of DEGs was performed using limma (R package, v3.2.3), with significant differences in gene expression in a single dataset being determined by p. value <0.05 and abs(logFC) > 1. Disease-associated genes were required to satisfy the requirement of being significant in three or more datasets, with the ANCA-AAV threshold set at two for only four datasets, exceptionally.

### 2.3 scRNA-seq data process and analysis

In the single-cell dataset, data were processed and analyzed using Seurat (R package, v3.2.3), and batch effect correction was performed using harmony (R package, v0.1.1). Significance thresholds in the single-cell analysis were set at p < 0.05 and avg_logFC > 0.25. The differential genes between immune-mediated kidney disorders and health from a single-cell perspective were determined to be significant either in the holistic condition or in more than two clusters.

### 2.4 Analysis of bulk RNA-seq mapped to scRNA-seq

Only four samples in the Bi dataset had information on the known response of ICB treatment, and the samples treated with atezolizumab + bevacizumab in IMmotion151 were mapped to the Bi dataset using Scissor (R package, v2.0.0) corresponding to the parameter’s alpha = 0.05 and cutoff = 0.25. For the results of the mapping only the cells with consistent response were retained, i.e., those whose outcome was the same as either response or non-response in the Bi. and in IMmotion151 cells were included in the downstream analysis. The mapped cells were still analyzed for DEGs between the response vs. non-response groups using Seruat, and significant DEGs were identified using p. value <0.05 and avg_logFC > 0.25.

### 2.5 Pathway and immune cell annotation and selection

All significant genes in each dataset were enriched pathways using the enrichPathway function from ReactomePA (R package, v1.30) and the profiling of 22 immune cells computed by CIBERSORT (Bulk RNA-seq) and SCINA (scRNA-seq), respectively. Autoimmune nephropathy-associated pathways need to meet the above criteria consistent with autoimmune nephropathy genes. Differential immune cells were screened in bulk using the Mann-Whitney U test and in scRNA-seq using the Fisher’ test. Immune cells associated with kidney disease also needed to meet the following criteria: (i) significant in three or more bulk datasets; (ii) significant in single-cell conditions; and (iii) significant in either bulk or scRNA for the same disease.

### 2.6 ICB data preprocessing

The log2FPKM matrices of IMmotion151, IMmotion150, JAVELIN, and PCD4989g were individually 0.75 quantile normalized, and combined with the CheckMate data, followed by batch correction using the ComBat function in the SVA (R package, v3.34). The expression matrix of genes for pre-feature selection was subset to autoimmune nephropathy-related genes. Pathway expression matrix analysis was performed using the ssGESA (GSVA, R package, v1.34) and subset to immune-mediated kidney disease pathways.

For the mutation matrix of all ICB datasets, only non-synonymous mutations were taken into account in this study. Candidate single-mutation variables that were significantly related to response (Fisher’ exact test, p. value <0.05) and survival (Cox-proportional hazards regression model, Wald test, p. value <0.05) in IMmotion151. Co-mutation genes are defined as pairs of genes that have either mutated or not. We filtered candidate co-mutation variables by response or survival in IMmotion151 and retained only those that were significantly associated with overall survival with KIRC in TCGA.

### 2.7 Feature selection and modeling

Five model variables require feature selection for subsequent analysis: genes expression, pathways expression, immune cell proportion, genes mutation, and co-mutation. Recursive feature reduction using caret (R package, v6.0-93) with 10-fold cross-validation was performed on all candidate variables. ML models were created using a variety of algorithms, including support vector machine (SVM), Naïve Bayes (NB), random forest (RF), k-nearest neighbors (KKNN), AdaBoost Classification Trees (AdaBoost), eXtreme Gradient Boosting (XGBoost), Neural Network (NeuralNet). The caret package (R package, v1.7.5.1) provides the first five methods, nerualnet package (R package, v1.44.2) provides the NeuralNet algorithm, and XGBoost obtain from the xgboost package (R package, v6.0-93).

### 2.8 Comparsion for different multi-gene models

To evaluate the performance of the models, we compared it with the ICB-derived gene model and biological functional characterization models sequentially. For ICB-derived genes selected by: (i) top 25 response significant genes (TopRes); (ii) top 25 survival significant genes (TopPFS); (iii) combined top 25 significant genes in response and survival (survival and response multiplied by p. value and sort in ascending order then took the top 25 genes, TopSig). The ICB-derived model built by XGBoost were identical to the TIs-ML model in procedure. The set of ICB-related functional gene lists were collected from multiple studies and is detailed. Expression scores for above functional signatures were calculated by the ssGSEA function from GSVA (R package, v1.34), and the pROC (R package, v1.18) was used to generate the ROC curves by calculate the sensitivity and specificity of the scores in predicting the ICBs’ binary responses. Other ICB response prediction model included that: the GEP score were computed by 18 immune genes expression which formula provided in Keynote-028 (Ott et al., 2019); the TME score were computed by TMEscore (R package, v0.1.4); TIDE score was computed by the TIDEpy python package (https://github.com/liulab-dfci/TIDEpy).

### 2.9 Identifying clusters of autoimmune nephropathy associated genes

716 genes were identified in the immune-mediated kidney disorders samples from the above analytic processes and excluded the housekeeping genes. We clustered the expression matrix of autoimmune nephropathy genes of IMmotion151 using ConsensusClusterPlus (R package, v1.50), resulting in two distinct consensus that cluster by k-means and distance compute by Euclidean. The cosine similarity was determined by calculating the top 50 genes that were specifically highly expressed in each cluster. For the assignment of the CheckMate and JAVELIN sample clusters, greater cosine similarity with the known IMmotion151 clusters was labeled corresponding clusters (a similarity lower than 0.4 would assign in NA).

### 2.10 Statistical analyses

R v3.6.3 (https://www.r-project.org) was used to perform all statistical analyses. The two-sided Mann-Whitney U test was used to compare the difference in scores between the response and non-response (as well as other clinical) groups. Fisher’s exact test was utilized to determine whether there is a significant correlation between discrete variables and clinical groups. The logarithmic ranking test was used to assess prognostic differences between subgroups of clinical or model scores for Kaplan-Meier (KM) survival curves. Adjusted p. value of DEGs and GSEA analysis computed by the Benjamini-Hochberg method. By default, continuous variables greater than the median or quantile of 0.75 would be classified as high. The correlation between model score and immunological characteristics or cancer hallmarks was evaluated using Spearman correlation.

### 2.11 Data available

All immune-mediated disorders clinical and transcriptome data were collected from Gene Expression Omnibus (GEO) database (Edgar et al., 2002), except a scRNA data of LN, it’ processed data were from https://immunogenomics.io/ampsle, https://immunogenomics.io/cellbrowser/ and https://portals.broadinstitute.org/single_cell/study/amp-phase-1. For immuno-cohorts, clinical and sequencing data of IMmotion151, as well as IMvigor210, POPLAR, IMmotion150, and PCD4989g, were obtained from the EGA dataset with study IDs of EGAC00001001813 and EGAS00001004343, while CheckMate (Braun et al., 2020), JAVELIN (Motzer et al., 2020a) were from Supplementary Materials. Mutation and clinical data of ccRCC from The Cancer Genome Atlas (TCGA) database were downloaded from cbioportal (https://www.cbioportal.org/study/summary?id=kirc_tcga). Details see Supplementary Data S1.

## 3 Result

### 3.1 Overview of TIs-ML method for ICB predictions

**FIGURE 1:**
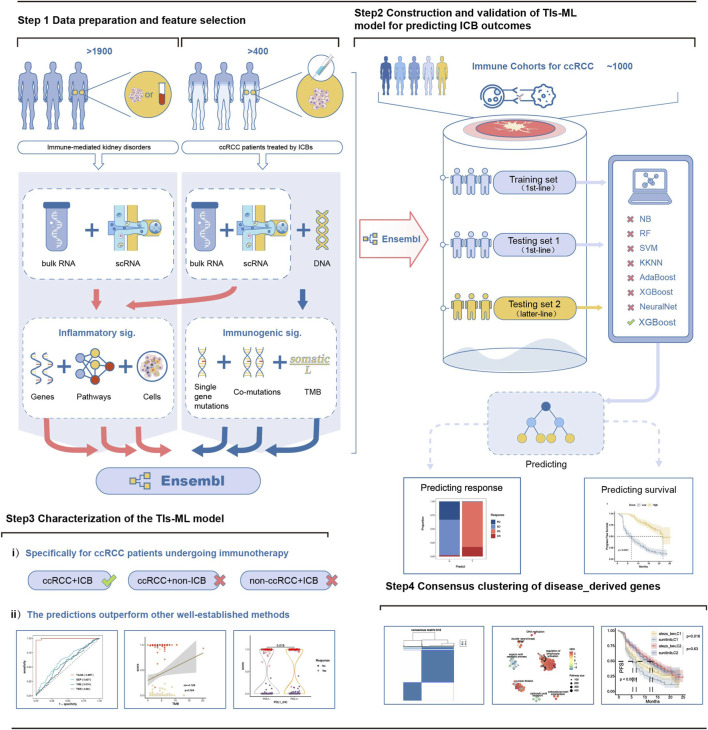
Conceptual workflow of this study and development of the TIs-ML model. Step 1: Data preparation and feature derivation. We integrated datasets from >1,900 patients with autoimmune kidney diseases and >400 patients with clear cell renal cell carcinoma (ccRCC) prior to undergoing immune checkpoint blockade (ICB) therapy. Bulk RNA-seq, microarray data, scRNA-seq, and whole-exome or targeted sequencing were used to generate transcriptomic and genomic profiles. From these datasets, six categories of tumor–immune (TI) features were derived, including inflammatory signatures (gene-level, pathway-level, and immune-cell signatures) and immunogenic signatures (single-gene mutations, co-mutation patterns, and tumor mutational burden), which together formed the ensemble TI feature matrix. Step 2: Model construction and validation. The model input encompassed inflammatory expression signatures of genes, pathways, and immune cells, as well as immunogenic mutation signatures of single gene mutations, co-mutations, and tumor mutation burden (TMB). The ensemble of features was utilized to construct an XGBoost machine learning (ML) model. We trained a series of machine-learning classifiers. An ensemble framework was used to build the final TIs-ML model. Model performance was evaluated in independent first-line and later-line cohorts for prediction of ICB response and survival outcomes. Step 3: Functional characterization of the TIs-ML model. (i) The model was systematically assessed across ccRCC+ICB, ccRCC+non-ICB, and non-ccRCC+ICB groups to confirm disease- and treatment-specificity. (ii) Comparative analyses demonstrated that the TIs-ML model outperformed several commonly used clinical and molecular predictors. Step 4: Consensus clustering of disease-derived genes. Autoimmune nephropathy–derived TI genes were subjected to consensus clustering to identify biologically meaningful subgroups, followed by functional annotation, immune landscape comparison, and survival analyses.

Previously published works suggested a connection between the effectiveness of immunotherapy and autoimmune diseases (Grandi and Tramontano, 2018; Masetti et al., 2021). Figure 1 provides an overview of the conceptual workflow of our study. We adopted five autoimmune nephropathies (Kant et al., 2022; Boesen and Kakalij, 2021) with scRNA or RNA-seq data. Ultimately, we investigated a total of 42 cohorts, including eight lupus nephritis (LN) cohorts, nine IgA nephropathy (IgAN) cohorts, twelve membranous nephropathy (MN) cohorts, nine focal segmental glomerulosclerosis (FSGS) cohorts, and four antineutrophil cytoplasmic antibody vasculitis (ANCA-AAV) cohorts, which were over 1,900 samples. We obtained a total of five cohorts of ccRCC with ICB treatment, which included over 1,000 patients.

To investigate the association between inflammatory signatures of autoimmune nephropathy and immunotherapy, we analyzed the expression array data of patients with LN (GSE32591_glo) (Be et al., 2012). In total, we discovered 649 differential pathways and 30 clusters when comparing LN patients to healthy controls for differential expression analysis (Supplementary Figure S1). Macrophage, and interferon-related pathways were been identified, and those biomarkers have been shown to have a strong correlation with irAE (Jing et al., 2020). The results revealed that “regulation of leukocyte mediated immunity” was the most upregulated pathway class in LN, along with “regulation of viral genome replication” and “regulation of defense response to virus by host”, which are related to human endogenous retroviruses (HERVS). The results suggested that ccRCC was an inflammatory tumor, and inflammatory signals were connected to immunotherapy, which was consistent with previous studies (Hegde and Chen, 2020).

### 3.2 Inflammatory signatures were associated with ICB response for autoimmune nephropathy bulk RNA analysis

**FIGURE 2:**
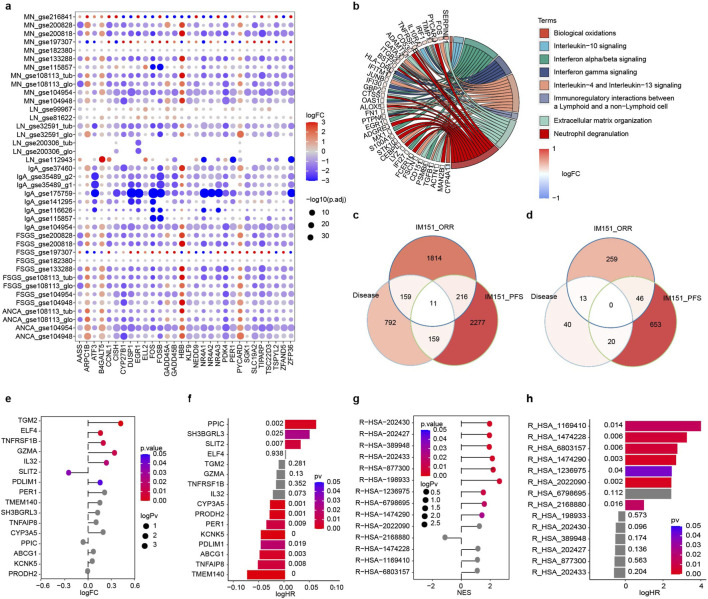
Inflammatory signatures of autoimmune nephropathy generated from bulk RNA data. **(a)** The top 30 Differentially Expressed Genes (DEGs) among kidney-mediated diseases between patients and healthy controls in each dataset. The x-axis represents the gene symbols, while the y-axis lists immune-mediated kidney illnesses and the corresponding datasets. Positive logFC represents upregulated in disease and negative the opposite. **(b)** Representative Reactome pathways and core genes altered in kidney-mediated disease expression in IMmotion151. Positive logFC represents upregulated in the responders’ group and negative the opposite. **(c, d)** Overlap of signatures from renal disorders and IMmotion151 with those associated with **(c)** genes and **(d)** pathways. **(e-h)** Lollipop charts display kidney disease-derived **(e)** genes and **(g)** pathways expressed differently in responders and non-responders of IMmotion151. Positive logFC represents higher expression in responders and negative the opposite. Bar graphs indicate the correlation of **(f)** gene and **(h)** pathway expression with progress-free survival (PFS) in IMmotion151. Positive logHR represents bad prognosis in higher expression whereas negative logHR represent good prognosis in higher expression.

Inflammation is a complication of autoimmune nephropathy, and inflammatory signatures produced by the immune system are related to the immune response. To investigate the connection between inflammatory signals and immune responses, we disaggregated different types of bulk RNA data from over 1,900 patients with immune-mediated kidney disease. After conducting a differential analysis between the cases and healthy controls within each dataset of five diseases, we obtained 1,121 differentially expressed genes (DEGs) in LN 55, IgAN 90, MN 586, FSGS 559, and ANCA-AAV 581 separately (Supplementary Figure S2a). We found that the expression patterns of the top 30 DEGs were generally consistent across distinct datasets, indicating that certain shared molecular processes may contribute to the etiology of diverse autoimmune disorders (Figure 2a and Supplementary Data S2). Genes were enriched in the frequent pathways, such as “interferon gamma signaling” pathway, “interferon signaling” pathway, etc., that are relevant to irAE and immune response (Hu et al., 2023; Head et al., 2019; Ayers et al., 2017). Among them, the immunoregulation-related gene EGR1 (Early Growth Response 1), involved in Interferon alpha/beta signaling, which is a typical pro-inflammatory cytokine (Pozhitkov et al., 2017) and enhances anti-tumor capacity by regulating and activating T cells (Dan et al., 2020), was differentially expressed in all of the five diseases (Figures 2a,b). We further evaluated the aforementioned signatures in the IMmotion151 dataset (Buttner et al., 2022), where 400+ previously untreated ccRCC patients were given atezolizumab (an anti-PD-L1 inhibitor) plus bevacizumab (an anti-VEGF inhibitor), and found 329 genes relevant to response and survival (Figure 2c). Inflammation is a major class of functions enriched for differentially expressed genes in the autoimmune nephropathy patients compared to healthy controls, and such genes show the potential efficacy to predict response or prognosis of ICB therapy.

Furthermore, pathway signatures can replenish the predictions by gene signatures. Within bulk RNA data, we totally enriched a total of 73 differentially expressed Reactome pathways (DEPs, Supplementary Figures S2b,c and Supplementary Data S3). Among them, 33 pathways relevant to response and survival in IMmotion 151 (Figure 2d). In particular, we focused on the canonical pathways most associated with the inflammatory response: interferon pathway; Interleukin pathway; TLR pathway; NF-kB pathway; TCR pathway; BCR pathway, as well as the PD-1 pathway and Apoptosis pathway which are directly related to ICB therapy. The results showed that most of the inflammation-related pathways were significant in at least two or more diseases, especially the TCR pathway and apoptosis pathway were significant in all diseases, and the interleukin pathway and PD-1 pathway were significant in four diseases other than IgAN (Figures 2e-h). Intriguingly, most of these signatures upregulated in responders as compared to non-responders, and prediction ability of pathway signatures were slightly superior to those of gene signatures (Figures 2e-h). Pathways further explain the changes in inflammatory expression patterns of autoimmune nephropathy from a functional perspective and can serve as a credible complementary variable in ICB prediction.

Immune cells may be the direct hub of the immunoreactions in both immune diseases and tumors. We employed CIBERSORT to assess the relative proportions of 22 immune cell types in each specimen (Supplementary Figure S2d). One of the most significantly differentiated cell types, monocytes, was highly concentrated in immune diseases compared to controls. The infiltration and accumulation of them closely related to the pathogenesis of immune diseases (Zheng et al., 2020; Tang et al., 2021), and they can also differentiate into dendritic cells to present antigens, or secrete a variety of cytokines to active T cells during tumor immunotherapy (Ugel et al., 2021) (Supplementary Figure S2d). In summary, our findings suggest that different levels of inflammatory biomarkers derived from bulk RNA data of autoimmune nephropathy can provide valuable insights for predicting ICB outcomes.

### 3.3 Expansion of inflammatory signatures through single-cell RNA analysis

**FIGURE 3:**
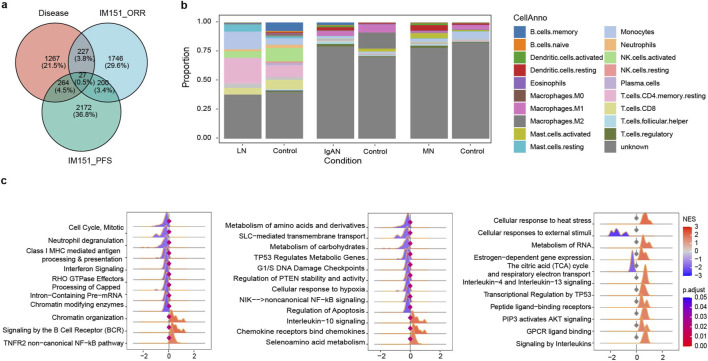
Inflammatory signatures of autoimmune nephropathy generated from scRNA data. **(a)** Overlap of gene signatures derived from kidney diseases with those related to response and prognosis in IMmotion151. **(b)** The stacked bar graphs of LN, IgAN, and MN demonstrate the percentage of 22 immune cells in the disease and control groups. **(c)** The ridge maps represent Differential Expression Pathways (DEPs) of the LN (left), IgAN (middle), and MN (right) datasets, which were obtained from the comparison between patients and healthy controls. Positive normalized enrichment score (NES) represents up-regulated in disease groups and negative the opposite.

Single-cell RNA sequencing can effectively complement bulk RNA sequencing through high-resolution expression data. We collected three single-cell RNA datasets (Supplementary Data S1) from LN, IgAN, and MN to perform a differential expression analysis. Specifically, we identified a total of 1,785 DEGs, which included 440, 1,264, and 616 genes in LN, IgAN, and MN, respectively (Supplementary Figure S3a). Compared with bulk RNA data, the scRNA-seq analysis reported 1,477 new DEGs, and only 308 (17.3%) DEGs were shared (Supplementary Data S4). This result confirmed that scRNA-seq was capable of complementing signatures at single cell levels. The DEGs identified in scRNA alone were mainly enriched in the “Immune System (HSA-168256)” and “Innate Immune System (HSA-168249)” (Supplementary Data S5). Moreover, “interleukin signaling” pathways associated with genes was upregulated, which turned out to be closely correlated with irAE (Jing et al., 2020) and was retrieved by scRNA-seq (Supplementary Figure S3d). In addition, a total of 518 DEGs were detected that were relevant to response and survival in the IMmotion151 dataset (Figure 3a). As a result, based on scRNA-seq data, more inflammatory genes involved in immunotherapy may be distinguished.

Furthermore, the results of DEPs through scRNA-seq data analysis were largely consistent. We additionally discovered 182 DEPs that were associated with autoimmune nephropathy, and 145 of these DEPs had inconsistencies with those discovered through bulk RNA datasets (Supplementary Figure S3b). DEPs, which were only remembered in scRNA data, were also crucial for immunoregulation and immunoreaction in malignancies. For example, “DDX58/IFIH1-mediated induction of interferon-alpha/beta (R-HSA-168928)” is an inflammation-related signal but is associated with immune response in tumor immunotherapy based on recognition of intracellular viral RNA and activation of interferon-stimulated gene expression, et al. (Yoneyama and Fujita, 2007; Loo et al., 2008; Honda et al., 2005) It was effective in distinguishing immune responders from non-responders (Supplementary Figure S3e). Above all, 66 pathway signatures were found using scRNA analysis in the IMmotion151 dataset, which was two times the number of those produced from bulk RNA data (Supplementary Figure S3c). Hence, scRNA-seq data enhanced the accuracy of results by revealing the differences resulting from the immune microenvironment.

Immune cells were annotated by SCINA in the scRNA-seq datasets (Zhang et al., 2019). We counted the immune cells within the 22 cell types that were associated with kidney diseases using Fisher’s exact test with p. value <0.05 (Figure 3b; Supplementary Figures S3f–h). T. cells.CD4. memory.resting was shown to have significantly lower levels in three disease groups comparing to healthy controls (Supplementary Figures S3f–h), which was consistent with previous reports (Okoye and Picker, 2013).

Eventually, we combined bulk and scRNA signatures by the principle: i) it must become significant in the relevant disease; ii) it in scRNA is needed to be significant generally or in at least two clusters; iii) it will be selected if significant in bulk or single-cell for each disease. As a result, we integrated 1,016 genes, 93 pathways, and 16 immune cells of inflammatory signatures from autoimmune nephropathy (Supplementary Figures S3i–k and Supplementary Datas S4, S5). In summary, our test emphasized the importance and necessity of adding single-cell RNA data to bulk RNA for inflammatory biomarkers exploring.

### 3.4 Integrative analysis of inflammatory signatures for predicting immunotherapy response

**FIGURE 4:**
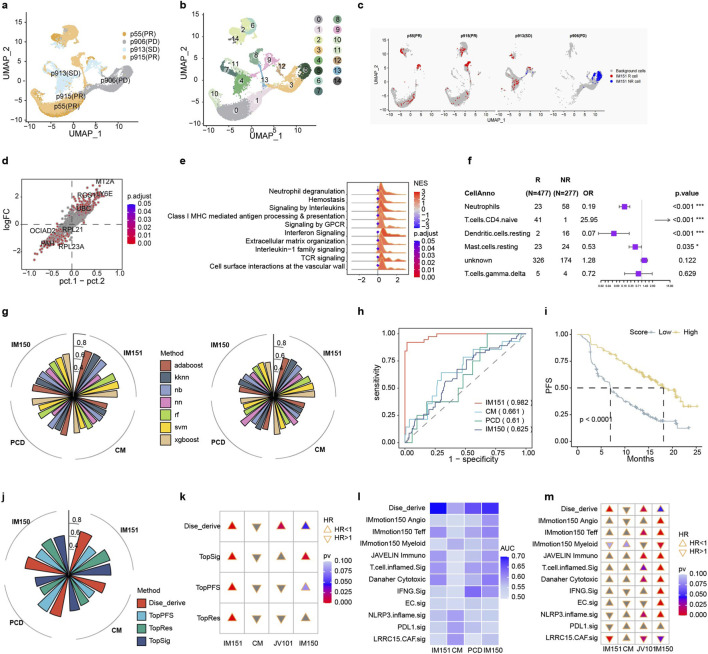
Cell mapping and RNA model generation. **(a, b)** Uniform Manifold Approximation and Projection (UMAP) plots, which show the cell clusters from four patients in Bi et al. (2021). **(c)** UMAP plots for the bulk mapping results for each patient. In these plots, responders and non-responders of IMmotion151 mapped to Bi display red dots and blue dots correspondingly. **(d–f)** Results of differential analysis from mapped effective and ineffective cells, differentially expressed genes (DEGs, **(d)**), Differential Expression Pathways (DEPs, **(e)**), and differentially immune cells (DICs, **(f)**). **(g)** Radar maps that compare the AUC of seven machine learning models when gene signatures (left) and pathway signatures (right) from four different ccRCC immune cohorts are used as input. Positive logFC and NES represent higher expression in responders than non-responders and negative the opposite. **(h)** Receiver Operating Characteristic (ROC) curves for the integrated inflammatory features models that evaluated in four ICBs datasets. **(i)** Kaplan-Meier progress-free survival (PFS) curves of inflammatory features models score in IMmotion151. P. value represents the significance of the log-rank test. **(j, k)** Efficacy comparisons between model derived from immune-mediated kidney disorders (Dise_derive) and model derived from ICBs. AUC for predicting the response **(j)** and Cox proportional hazards survival test **(h)** in four datasets. The hazard ratio (HR) bigger than 1 in the heatmap represents a bad prognosis for the higher score group. **(l, m)** Efficacy comparisons between dise_derive and bio-functional signatures collected from published studies. AUC for predicting the response **(l)** and Cox proportional hazards survival test **(m)** in four datasets.

Obtaining immunopredictive markers in ccRCC patients undergoing immunotherapy may enhance the specificity of immune response predictions. Additionally, the confidence of the indicators can be improved by combining bulk and single-cell RNA data from immunotherapy. Therefore, we mapped the bulk RNA expression data of IMmotion151, divided according to responders (complete response and partial response; CR + PR) and non-responders (stable disease and progressive disease; SD + PD), to the scRNA-Seq data obtained from Bi et al. (2021), which included four patients treated with anti-PD1-based therapies (Figures 4a,b; Supplementary Figure S4a). This combination approach of bulk and single cells revealed a strong correlation within the responders between the two datasets (Figure 4c) as well as the two PR patients from Bi et al. (2021) (Supplementary Figure S4b; Pearson correlation = 0.84, p. value = 9.93e-05). Cells in Bi. that had consistent response status (responders or non-responders) with IMmotion151 were kept for further study. Additionally, we got 1711 DEGs and 240 DEPs involving the immune system, hemostasis, extracellular matrix organization, and signal transduction, between mapped effective and ineffective cells. (Figures 4d,e, and Supplementary Datas S4, S5). MT2A, a member of the metallothionein gene family, was obviously upregulated in responders but barely expressed in non-responders, which is consistent with previous findings (Deng et al., 2022), hinting at a potential immuno-predictive biomarker (Figure 4d; Supplementary Figure S4c). We further explored DICs using a similar approach to that used in kidney diseases (Figure 4f; Supplementary Figure S4d) and tested the prediction ability of these gene and pathway signatures within the IMmotion151 cohort (Supplementary Figures S4e–h). The aforementioned findings imply that the immunopredictive markers discovered using the mapping approach exhibit a strong correlation with the prognosis of ccRCC patients undergoing immunotherapy.

The mapped DEGs in Bi et al. were intersected with the autoimmune nephropathy DEGs of bulk and scRNA respectively. The genes common to these two groups were merged, and housekeeping genes were excluded. Protocol of DEPs process were same to DEGs. Finally, 716 genes and 92 pathways specific to immune-nephrosis were obtained. Incorporating these markers as intersections with those discovered in the context of renal immune diseases can effectively reduce the number of variables while enhancing the level of confidence. Eventually, 25 genes, 47 pathways, and four immune cells were reserved after feature selection by Recursive Feature Elimination (RFE) in the IMmotion151 training datasets (Supplementary Data S6).

Next, we selected seven ML models to compare AUC for multi-genes and multi-pathways individually. XGBoost obtained consistent predictions for response and prognosis by comparing several methods, which was retained for more in-depth study (Figure 4g; Supplementary Figures S4i–l). We created an ensemble model using XGBoost based on the models of genes, pathways, and immune cells. And the combined inflammatory signatures ML model could make a robust prediction for ICB response and progress-free survival (PFS) in training data from IMmotion151 (Motzer et al., 2022) (IM151), as well as from four additional independent cohorts: CheckMate (Braun et al., 2020) (CM), JAVELIN (Motzer et al., 2020a) (JV101), PCD4989g (Herbst et al., 2014) (PCD), and IMmotion150 (McDermott et al., 2018) (IM150). The inflammatory ensemble model was able to achieve an AUC of 0.98 in the predictive validation set IMmotion151 (Figure 4h), and the model’s scores were significantly able to differentiate PFS in IMmotion151 at p. value <2.18e-20 (Figure 4i). The performance of the inflammatory model continued to be noteworthy in the independent validation set, with AUCs greater than 0.6 and survival log-rank test significantly in both JAVELIN and IMmotion150 (Figure 4h; Supplementary Figure S4). In all datasets, groups with higher scores tended to have a better prognosis than those with lower scores (Supplementary Figures S4m–o).

To compare the model efficacy of ccRCC with ICB-derived models and immune-mediated kidney disorders-based models, we build (i) ORR (TopRes); (ii) PFS (TopPFS); (iii) doubly significant genes (TopSig) models (see Method for details). The results indicate that disease-derived (Dise_deriv) gene models perform more effectively and have higher robustness. However, the generalization ability of ICB-derived models’ performance was suboptimal (Figure 4j and Supplementary Data S7). In terms of prognosis prediction, the disease-derived model discriminates prognosis in three datasets, whereas the best ICB-based model, the doubly significant gene model (TopSig), can only discriminate prognosis in two datasets (Figure 4k).

To determine whether diseased-derived models demonstrate superior predictive validity over other biologically sourced models, we compared it to 11 published genetically relevant models (Supplementary Data S8). The results demonstrate that disease-derived models were still the most effective at predicting both response and prognosis, while other models were less effective or could solely predict prognosis but not response (Figures 4l,m). For instance, LRRC15. CAF.sig (Dominguez et al., 2020) could predict the prognosis among IMmotion151, JAVELIN, and IMmotion150, but response predictive efficacy AUC was all lower than 0.6. In conclusion, the model based on inflammatory biomarkers is obviously superior to immunotherapy-based signatures models or published features with tumor biology, both in distinguishing immune response and survival for ccRCC with ICB.

### 3.5 Limitation of immunogenic signatures using genomic data for ICB response assessment

**FIGURE 5:**
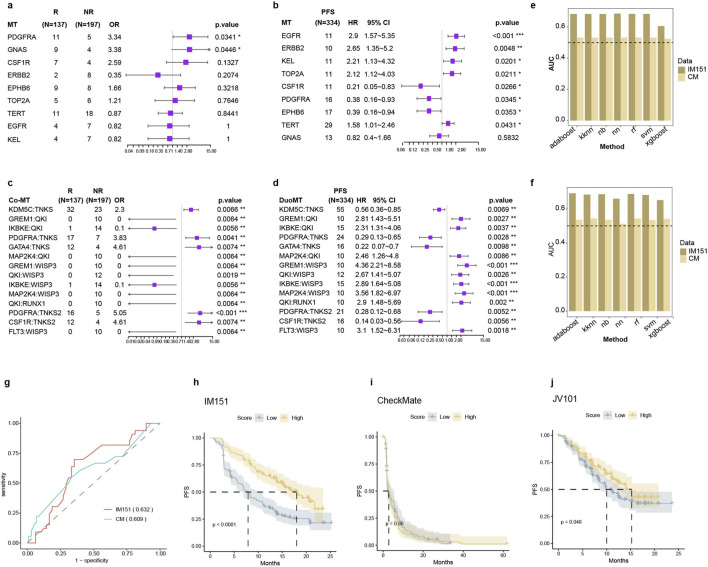
Immunogenic Signatures-Based Predictive Model for Immunotherapy Outcomes. **(a–d)** depict the predictive values of single gene mutations for **(a)** response and **(b)** survival outcomes, as well as co-mutations for **(c)** response and **(d)** survival outcomes. **(e, f)** AUC comparison of seven distinct machine learning models, with the result of single gene mutations and **(f)** co-mutations in immunological cohorts. **(g)** ROC curves for the integrated immunogenic signature models that were evaluated in the two DNA data available ICBs datasets. **(h–j)** Kaplan-Meier survival curves of immunogenic signatures models score in IMmotion151 **(h)** CheckMate **(i)** and JAVELIN **(j)**.

Immunogenic biomarkers are critical in assessing the immune response. However, the efficacy of using a single biomarker to predict the response to immunotherapy in ccRCC is constrained. The calculation of TMB, which includes all nonsynonymous mutations, demonstrated modest predictive capacity with an AUC of 0.572 in the IMmotion151 cohort (Supplementary Figures S5a,b). Using Fisher’s exact test and Cox proportional hazards regression analysis, we identified genes that were differentially mutated in response or prognosis with ICB. In the end, 14 mutation genes were selected as potential immunogenic signatures (Figures 5a,b).

Notably, the highest population frequency of the concurrent mutation gene pairs, EPHB6-GNAS, only in three patients (Supplementary Figure S5c). We calculated co-mutation genes, which are pairs of genes that either one of them were mutated. Hence, we screened for co-mutated gene pairs in the IMmotion151 cohort by analyzing the differences in immune response and prognosis. In total, we identified 1,876 combinations of gene pairs (Figures 5c,d). To reduce selection bias, we filtered additional co-mutation genes based on the overall survival of The Cancer Genome Atlas (TCGA) in KIRC. Ultimately, we discovered 49 co-mutation genes associated with response and survival in patients.

We compared seven different approaches to building machine learning models based on multigene and co-mutation genes. XGBoost consistently demonstrated satisfactory performance in predicting response and prognosis using the IMmotion151 or CheckMate cohorts (Figures 5e,f; Supplementary Figures S5d–g). Overall, predictive models based on the three individual types of immunogenic signatures have demonstrated limited ability to estimate immune response and survival (Figures 5e,f; Supplementary Figures S5d–g). Finally, we established a machine-learning model that integrates all the immunogenic biomarkers. The model achieved an AUC of 0.632 and 0.609 in the testing set IMmotion151 and the independent validation set CheckMate cohort, respectively (Figure 5g). The comprehensive model’s predictive score was capable of stratifying the prognosis of different cohorts (Figures 5h–j). These results revealed that the single immunogenic model was inadequate, and the multi-indicator combination model improved the predictive efficiency of immunotherapy.

### 3.6 Integration of TIs-based machine learning tests for ICB effectiveness prediction

**FIGURE 6:**
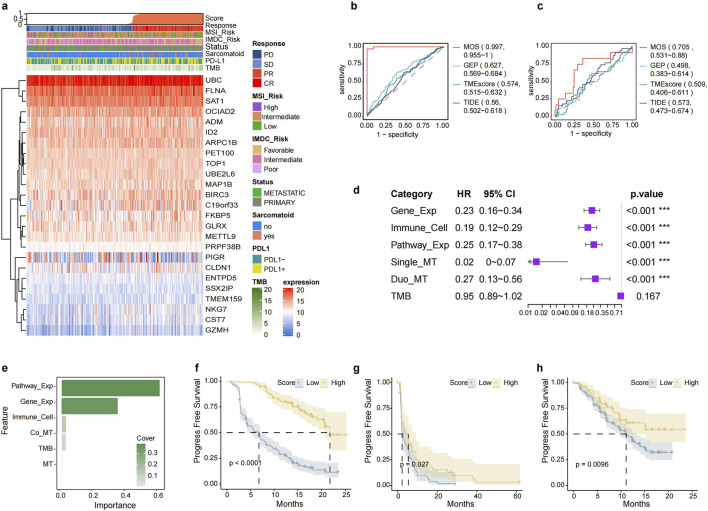
Multi-Omics model for predicting immunotherapy outcomes. **(a)** Expression profiles of 25 model gene signatures in the IMmotion151 dataset are depicted, representing a comprehensive view of the landscape. **(b, c)** ROC curves of response prediction efficacy comparison between multi-omics (TIs) model and previous immune-related ICBs prediction model in IMmotion151 **(b)** and CheckMate **(c)**. **(d)** Forest map of prognostical efficacy of each TIs signature for survival in IMmotion151 **(e)** The importance coefficient of each signature in contributing to the overall mode is illustrated. **(f–h)** Kaplan-Meier curves in the cohorts of **(f)** IMmotion151, **(g)** CheckMate, and **(h)** JAVELIN demonstrate the effectiveness of the TIs approach in predicting PFS.

The inflammatory model was more efficient than immunogenic model, but there’s still room for improvement in the inflammatory model. Two models derived from the distinct perspective and integration of the two may further enhance the predictive efficiency. We aimed to develop a machine learning model using multi-omics data to predict response in ccRCC patients receiving ICB treatment. The multi-omics signatures landscape were shown in Figure 6a, Supplementary Figures S6a,b, Supplementary Datas S9, S10. The ML-based multi-omics model was constructed using 354 patients with ICB from IMmotion151 who had RNA-seq and DNA data. The patients were randomly assigned into a training and validation set with a ratio of 2:1. Our TIs-ML model achieved an excellent AUC of 0.997 for distinguishing responders from non-responders in the validation set, significantly surpassing the predictive power of GEP score (AUC: 0.627), TME score (AUC: 0.574), TIDE score (AUC: 0.560), TMB (AUC: 0.572), and PD-L1 expression (AUC: 0.555) (Figure 6b; Supplementary Figure S5b). Positive findings were consistently observed when the model was applied to the CheckMate cohort; our model had an AUC of 0.705, whereas the GEP score had 0.498, the TME score had 0.509, and the TIDE score had 0.573 (Figure 6c). The present study suggests that the TIs-based predictive signatures are highly generalizable and that the TIs-ML model can robustly predict response to ICB.

In addition, individuals in the high-score group (with the threshold set at the median) in the IMmotion151 cohort had a longer PFS than those in the low-score group (Figure 6f; p. value < 0.0001). The CheckMate and JAVELIN101 cohorts (Figures 6g,h; Supplementary Figure S6c) showed a better prognosis in the high-score group (p. value = 0.027 for PFS and 0.54 for overall survival in the CheckMate cohort; p. value = 0.010 in the JAVELIN101 cohort). Similarly, The TIs-ML score outperformed typical biomarkers for predicting prognosis (Supplementary Figures S6d,e). These findings demonstrated that the TIs score could act as a prognostic biomarker for better patient stratification in ccRCC with ICB treatment.

More importantly, we revealed that inflammatory signatures demonstrated a higher importance coefficient than immunogenic signatures (Figure 6e) in the multi-omics model. Autoimmune nephropathy-related pathways and genes played a dominant role in the model (Figure 6d), suggesting that there was a strong correlation between immune-mediated kidney disease and immunotherapy.

### 3.7 Association of risk score with clinical factors and biological characteristics

**FIGURE 7:**
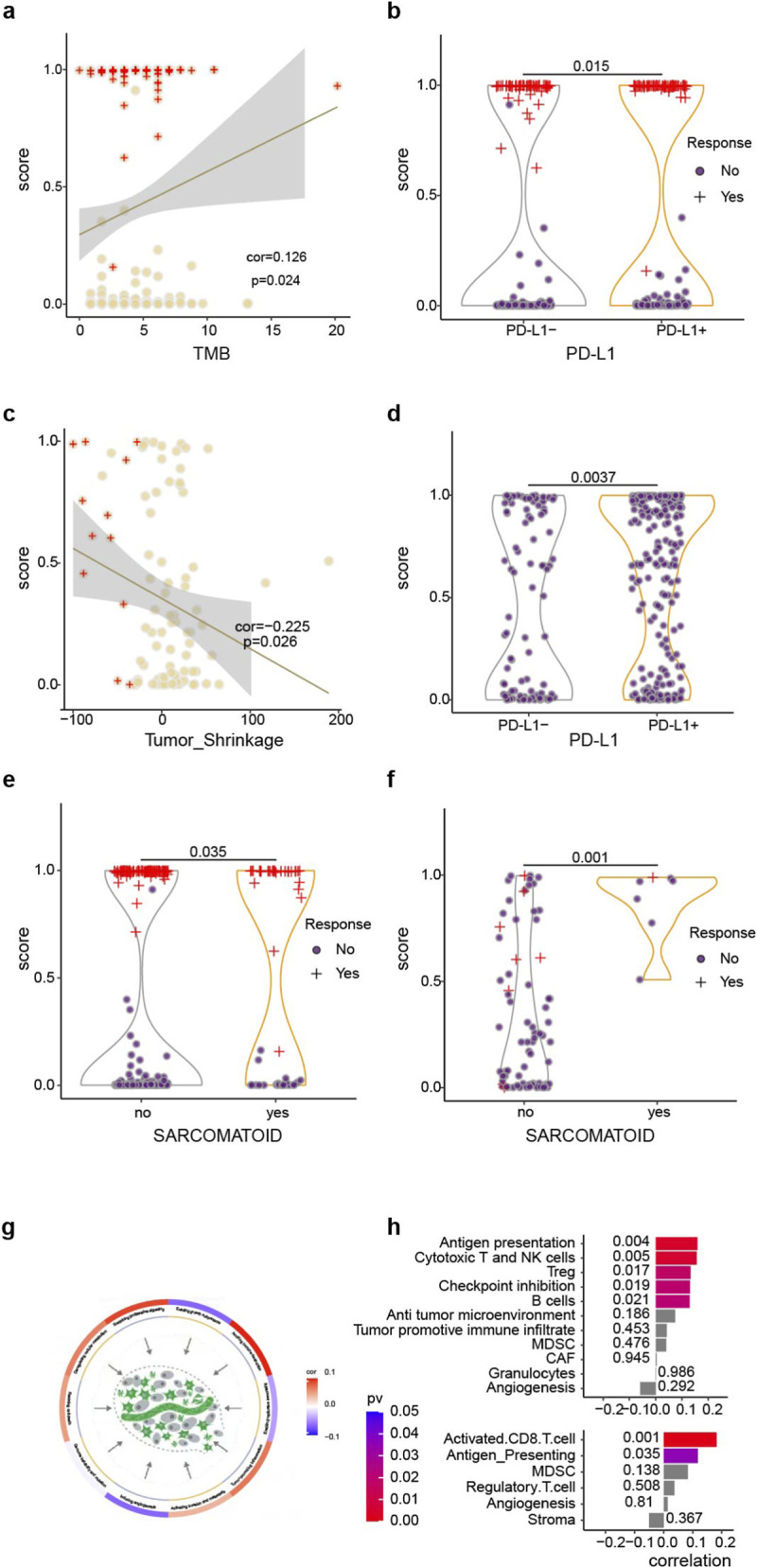
Correlations between the model score and various biological and clinicopathologic factors. The model score shows correlations with **(a)** tumor regression and **(c)** tumor mutation burden (TMB) in the IMmotion151 dataset. In addition, the model score is correlated with PD-L1 expression in both the **(b)** IMmotion151 and **(d)** JAVELIN datasets. Furthermore, the model score exhibits correlations with sarcomatoid differentiation in the **(e)** IMmotion151 and **(f)** CheckMates datasets. Moreover, the model score is correlated with tumor microenvironment-related signatures derived from **(g)** Bagaev.sig.2 and **(h)** Multi_Source.sig. The X-axis represents the correlations between TIs score and TME-related signatures.

We examined the correlation between the TIs-ML scores and clinical factors after observing a significant connection between the risk score and tumor regression (Figure 7a). A significant difference in TIs score was observed between sarcomatoid and non-sarcomatoid ccRCC, with sarcomatoid ccRCC tending to have a higher TIs score (Figures 7e,f). Our study also identified that the Memorial Sloan-Kettering Cancer Center (MSKCC) (Motzer et al., 1999) and the International Metastatic Renal Cell Carcinoma Database Consortium (IMDC) (Heng et al., 2009; Ko et al., 2015), which are commonly used to predict the prognosis in metastatic RCC using serological biomarkers, were less effective compared with TIs score (Supplementary Figures S7a,b,d,e). The results demonstrated that there was no significant difference in TIs score between primary and metastatic lesions (Supplementary Figures S7c,f), which emphasizes the robustness of our model in predicting response to ICB.

Furthermore, we explored the correlation between TIs scores and well-known immunotherapy biomarkers, including TMB and PD-L1. Responders were more closely related to high scores than high TMB (Figure 7c) and positive PD-L1 (Figures 7b,d). Besides, immunotherapy responders and survival-benefit patients with TMB-low or PD-L1-negative could be bailed by higher TIs-based risk scores, emphasizing that our multi-omics model can complement the prediction ability of conventional biomarkers (Supplementary Figures S7i,j).

We examined the association between risk scores and published ICB-related signatures, and identified a positive correlation between the risk scores and a variety of immune-related hallmark genes. The inflammatory response enhances the efficiency of immune checkpoint therapy by increasing immune cell infiltration and improving tumor immunogenicity (Supplementary Figures S7g,h). The study showed that the TIs score had a positive correlation with activated CD8 T cells and antigen-presenting signatures, as well as novel gene expression signatures such as NK cells, Treg, and B cells in the analysis of the TME (Figures 7g,h; Supplementary Figure S7h). These immune cell signatures are known to play a key role in the tumor-killing process.

### 3.8 TIs-ML was specific for ccRCC patients treated with immunotherapy

**FIGURE 8:**
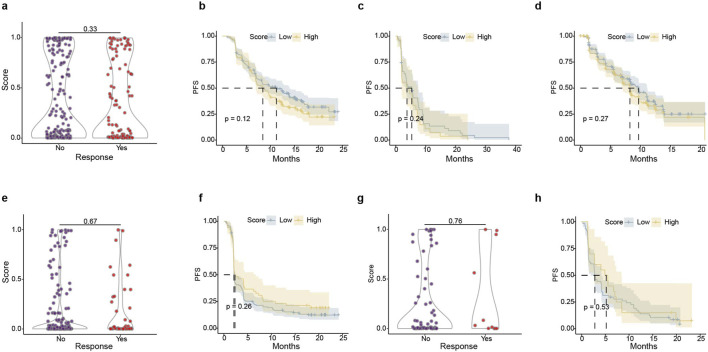
The multi-omics model is specifically designed for ccRCC patients undergoing immunotherapy. Our model cannot predict the responses of individuals treated with **(a)** sunitinib in IMmotion151 (ccRCC), **(e)** atezolizumab in IMvigor210 (mUC), and **(g)** atezolizumab in Poplar (NSCLC). Additionally, our model cannot predict the progress-free survival (PFS) of patients treated with **(b)** sunitinib in IMmotion151 (ccRCC), **(c)** everolimus in CheckMate (ccRCC), **(d)** sunitinib in JAVELIN (ccRCC), **(f)** atezolizumab in IMvigor210 (mUC), and **(h)** atezolizumab in Poplar (NSCLC).

Since the model score can predict the response and prognosis of immunotherapy in ccRCC, we tested the specificity of our model by evaluating non-immunotherapy treatment for ccRCC, and also by assessing immunotherapy treatment for other cancer patients. The risk score was ineffective in predicting response or prognosis between the single-agent targeted therapy and antiangiogenic therapy in ccRCC from the IMmotion151, JAVELIN, and CheckMate cohorts (Figures 8a–d). The IMvigor210 (mUC) (Balar et al., 2017) and Poplar (NSCLC) (Fehrenbacher et al., 2016) datasets were also analyzed using the ML-based inflammatory model with negative results for predicting prognosis or response (Figures 8e–h; Supplementary Figure S8). In conclusion, our method can specifically predict ICB outcomes in ccRCC patients, but is not suitable for predicting efficacy in ccRCC patients receiving other therapies or for individuals with other types of cancer undergoing immunotherapy.

### 3.9 Inflammation-associated differential expression gene in autoimmune nephropathy differentiating the result of immunotherapy

To further explore the roles and mechanisms of signatures derived from immune-mediated kidney disorders in guidance ICBs for ccRCC, biological function pathway enrichment analysis was performed for the disease-derived genes.

**FIGURE 9:**
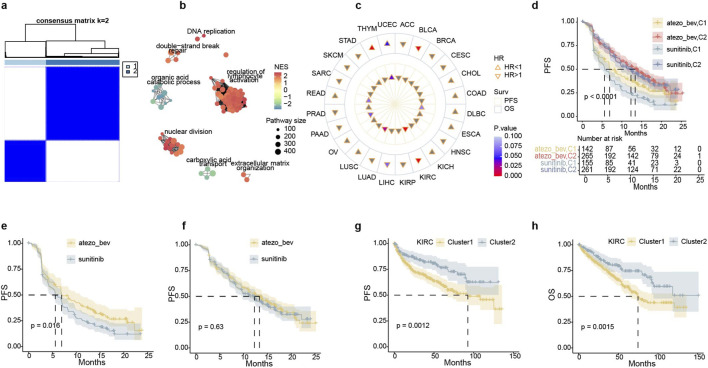
Consensus clustering of biomarkers obtained from immunological diseases. **(a)** Concensus clustering correlation heatmaps of IMmotion151 samples that genes associated with nephropathy were subjected to two consensus clusters. **(b)** Net graph of GO biological process pathways which significantly altered in Cluster 1 versus Cluster 2. Positive normalized enrichment score (NES) represents upregulated in Cluster1, and Negative NES represents downregulated in Cluster1. **(c)** Clusters prognostical effect on PFS and overall survival (OS) in TCGA pan-cancer cohorts. HR>1 represents that Cluster1 was the bad prognosis indicator compare with Cluster2 and HR<1 is the opposite. **(d)** Kaplan-Meier curves of the combination of treatment and clusters that demonstrated ICBs were superior to target monotherapy in cluster1. Clusters were significant in stratifying PFS **(e,g)** and OS **(f,h)** in IMmotion151 and TCGA KIRC, respectively.

Pathway enrichment analysis indicated that immune-mediated kidney disease genes have a strong association with biological processes, which plays a crucial role in immune stress and evasion in tumors (Figures 2e, 3c; Supplementary Figure S9a). Furthermore, we performed a consensus clustering analysis using these genes in the IMmotion151 dataset and identified two distinct clusters (Figure 9a). C1 and C2 differentially expressed genes were mainly enriched seven pathways, including “regulation of lymphocyte activation” and “nuclear division,” which plays a significant role in immunogenic cell death and exhibits high expression levels in C1 compared to C2 (Figure 9b). Interestingly, patients in Cluster 1 had a poorer prognosis. However, Immunotherapy improved PFS in the C1 patients while in the C2, neither immunotherapy nor any other therapy had an effect on the prognosis (Figure 9d; Supplementary Figures S9b–d). Consensus cluster analysis suggests that cluster1 patients are more suitable for monotherapy or combination therapy of ICB, while cluster2 could select more economical therapy in ccRCC. However, immune kidney disease signatures are likely only applicable to KIRC and may not be generalizable to most other cancer types in pan-cancer analysis (Figures 9c,e,f; Supplementary Figures S9e–h). In summary, the results demonstrate that kidney disease-related genes have a very strong correlation with immunotherapy at the molecular level and can specifically predict immunotherapy in renal cancer.

## 4 Discussion

In this study, our aim was to develop an immunotherapy predictive approach for all patients with ccRCC that is accurate, reliable, and precise. Our model is superior to the well-established biomarkers like TMB and PD-L1. Our model accurately and robustly predicted the response of ICB therapy, regardless of whether it was anti-PD1 or PD-L1 therapy, first-line or second-line therapy, or monotherapy or combination therapy.

We speculate that there may be connections between immune diseases and immunotherapy in tumors because cancer can suppress or escape the immune reaction and immunoreaction triggered by anti-PD-1 axis inhibitors may cause autoimmune diseases (Yasunaga, 2020). A preliminary experiment in LN vs. normal analysis demonstrated that immune kidney disease correlated with upregulated expression of “regulation of leukocyte mediated immunity” and pathways related to HERVS. Pathways enrichment clusters could correspond to the factors related to kidney ICB therapy. Notably, several features including interleukin-related signaling, extracellular matrix organization, and MHC-I antigen presentation pathways were biologically involved in shaping immunotherapy efficacy. These recurring features across datasets and models reinforce the functional relevance of inflammation and tissue remodeling in ccRCC immunobiology, supporting their role as potential therapeutic targets or biomarkers. Besides, we explained this hypothesis by consensus clustering analysis for nephropathy-related genes resulting that two clusters indicating distinct prognoses. Specifically, ccRCC patients in cluster 1 had poor prognoses but were more likely to benefit from combination programs of anti-PD-(L)1 plus multi-TKI (sunitinib). We were surprised that cluster 1 might not be a good indicator of immune monotherapy (Figures 9a,b), with a possible mechanism for sunitinib can enhance tumor infiltration by downregulating immunosuppressive cytokines and depleting Treg cells and MDCSs in murine models (Petroni et al., 2021). Our results suggest that the exploration of immune-predictive indicators from renal disease is on the right track.

Increasingly more and more study demonstrate that multi-omics features have advantages in accurately predicting outcomes. For example, Vanguri et al. integrated radiology, pathology, and genomics to predict ICB response in non-small cell lung cancer, reaching an AUC of 0.8, which outperformed unimodal of TMB (AUC = 0.61) and PD-L1 (AUC = 0.73) (Vanguri et al., 2022). Newell et al. combined genomics, methylomics, transcriptomics, and immune cell infiltrates to predict ICB response in melanoma an AUC of 0.84 (Newell et al., 2022). In our test, we incorporated bulk RNA profiles, single-cell RNA profiles, and genomics build the model to predict ICB response and prognosis in ccRCC, achieving excellent predictions efficacy across multiple cohorts. There is potential for reducing model complexity in the future. For example, the model could incorporate only a few robust gene expression markers, common driver mutations, and immune cell subpopulation infiltration markers while retaining acceptable predictive accuracy. By reducing the number of biomarkers, optimized compact panels could be designed to improve clinical feasibility and cost-effectiveness, thereby facilitating large-scale clinical utilization (Yang et al., 2025; Wang et al., 2025). With the rapid development of artificial intelligence-based histopathological image analysis, integration of molecular and pathology information is poised to improve predictive accuracy and reduce overall costs (Chen et al., 2024; Chen et al., 2022; Hoang et al., 2024).

However, our study has certain limits. All the cohorts employed were public datasets with a limited number of patients, and there was a lack of independent validation datasets from prospective clinical cohorts. Additionally, some patients may have been enrolled in crossover immunotherapy cohorts, which could lead to overfitting of the model results. In the next phase, we plan to recruit patients for the proposed immunotherapy, regardless of the number of lines of therapy or the treatment regimen. Multi-omics assays are both expensive and time-consuming for clinical use, making them a limitation. Alternative approaches are necessary to modify the variables to better align with clinical practice. Additionally, gene expression values are known to be less stable and can vary significantly among platforms, batches, and standardized forms. We explicitly acknowledged the challenge of clinical implementation due to cost, turnaround time, and the need for multi-platform assays. The complexity of indicator construction discourages the application of the model in clinical practice and necessitates further simplification of the model and reduce cost.

## 5 Conclusion

In this study, we developed a multi-omics features-based machine learning (TIs-ML) model to predict immune checkpoint blockade (ICB) response in clear cell renal cell carcinoma (ccRCC). By integrating inflammatory and immunogenic signatures from bulk RNA-seq, single-cell RNA-seq, and genomic data, our model outperformed established biomarkers like tumor mutation burden (TMB) and PD-L1 expression. The TIs-ML model demonstrated robust prediction accuracy across multiple cohorts, providing a novel method for guiding precise immunotherapy in ccRCC. Furthermore, our findings highlight the importance of integrating multi-omics data to improve immunotherapy outcome prediction. Overall, this study presents an innovative approach that utilizes multi-omics features for enhanced prediction of immunotherapy response and survival in ccRCC.

## Data Availability

The original contributions presented in the study are included in the article/Supplementary Material, further inquiries can be directed to the corresponding authors.
