# Supplementary material for: A multi-omics features-based approach integrating immunogenicity and inflammation enhances immunotherapy benefit in clear cell renal cell carcinoma
Source: Front Cell Dev Biol. 2026 Jan 20;13:1596719. doi: 10.3389/fcell.2025.1596719 (PMC12864441; doi:10.3389/fcell.2025.1596719)
Supplement: Supplementary file 1 [file Presentation1.zip › Supplementary material presentation/Supplementary_Material_Figures.docx]

Supplementary Material

##
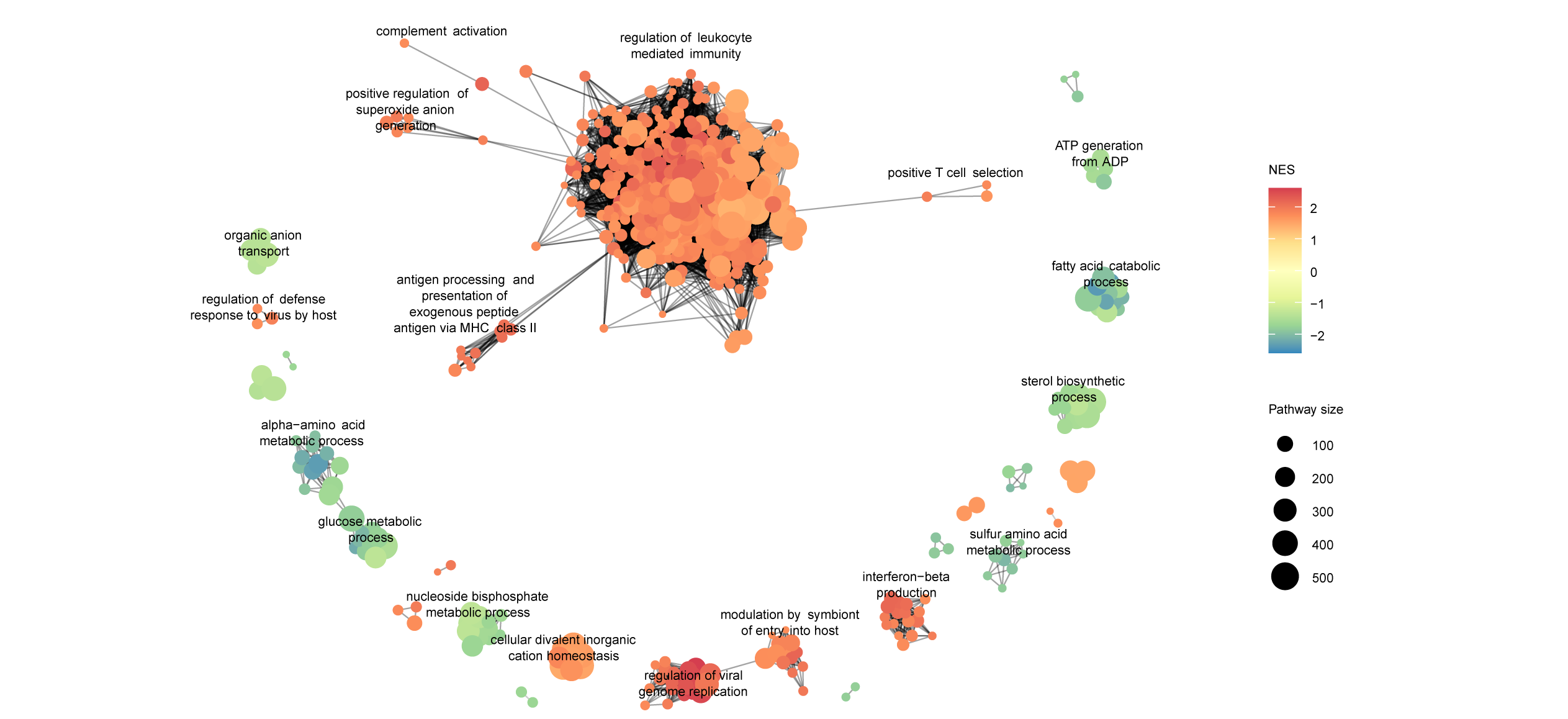
Supplementary Figures

## **Supplementary Fig S1.** Net graph of clusters from significant GSEA BP pathways generated by differential expression analysis between disease and control groups in the GSE32591_glo (lupus nephritis, LN) dataset. Positive normalize enrichment score (NES) represent pathways up-regulated in disease group. The largest clusters in LN were regulation of leukocyte mediated immunity and were in active expression compare with normal control.

**Supplementary Fig S2.** **Overlap of Differentially Expressed Genes (DEGs), Differentially Expressed Pathways (DEPs), and Differentially Immune Cells (DICs) across different types of nephropathies.** The Venn diagram illustrates the overlap of (a) DEGs and (b) DEPs between each type of nephropathy and healthy controls. (c) The top 30 DEPs and (d) the 22 DICs among kidney-mediated diseases across patients and healthy controls are depicted. The y-axis displays immune-mediated kidney illnesses and their associated datasets, while x-axis represents the reactome pathway names (c) and immune cells (d). Count was the significant DEGs number located in the corresponding pathway. logFC represent the logarithmic fold change between normal and disease that positive logFC demonstrate up-regulated in disease and negatibe logFC demonstrate down-regulated in disease.


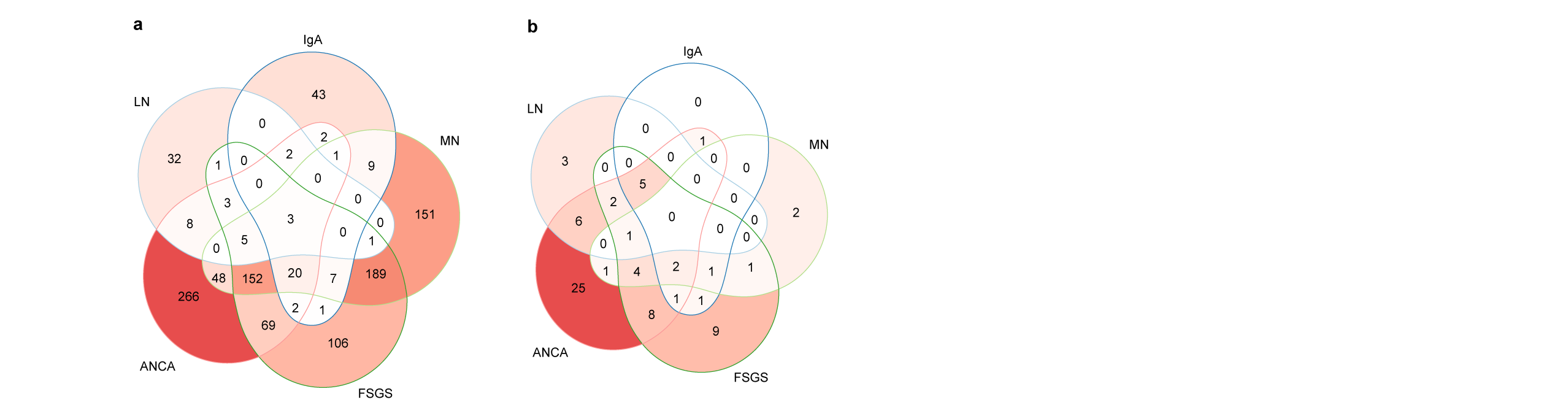


**c**

**d**


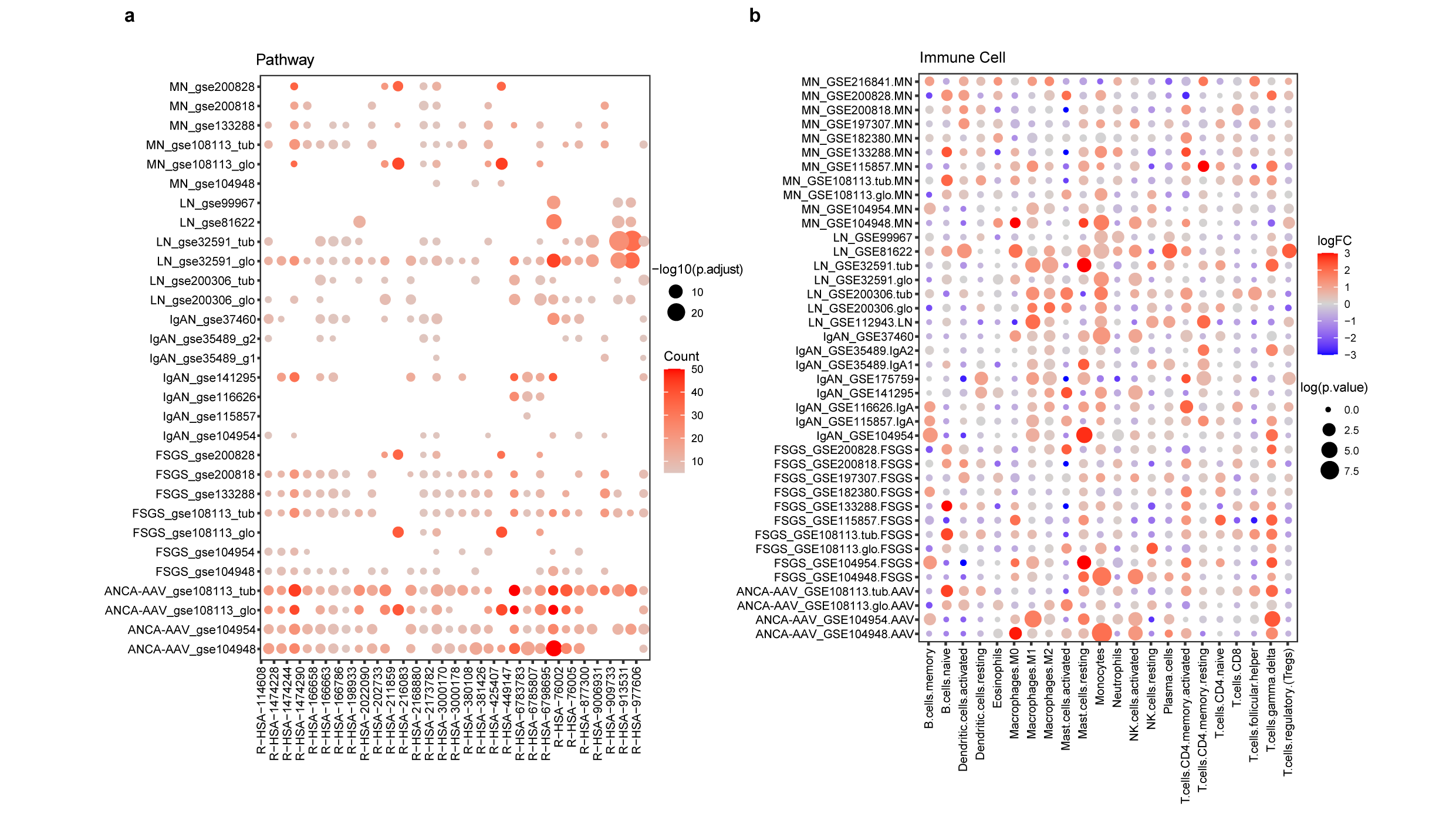

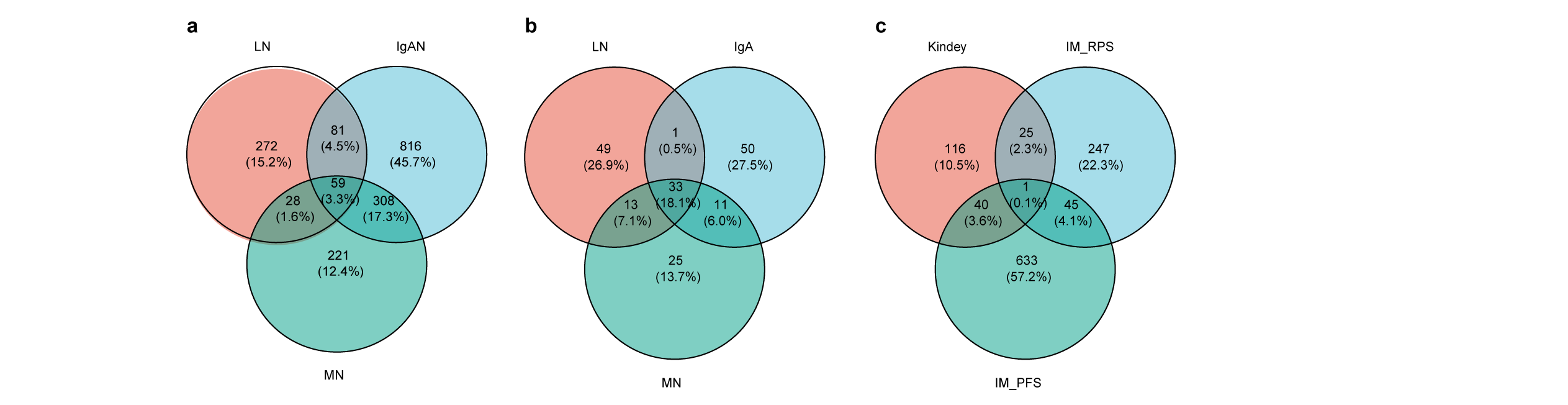

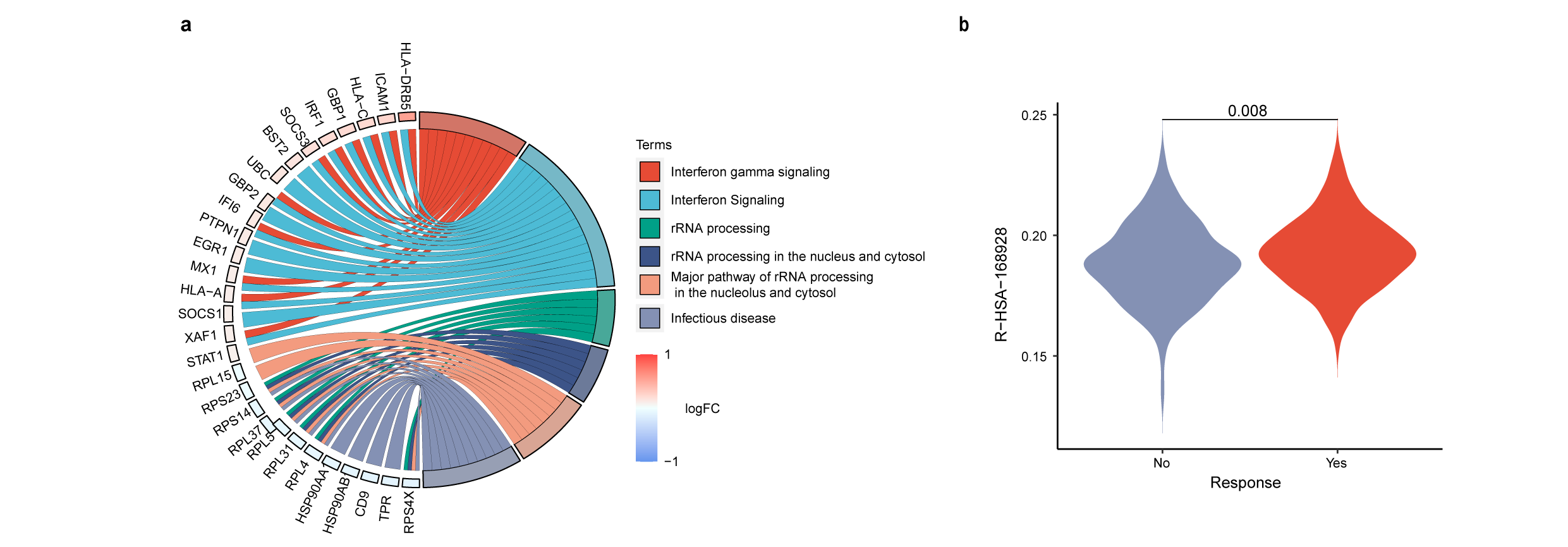


**d**

**e**

**f**


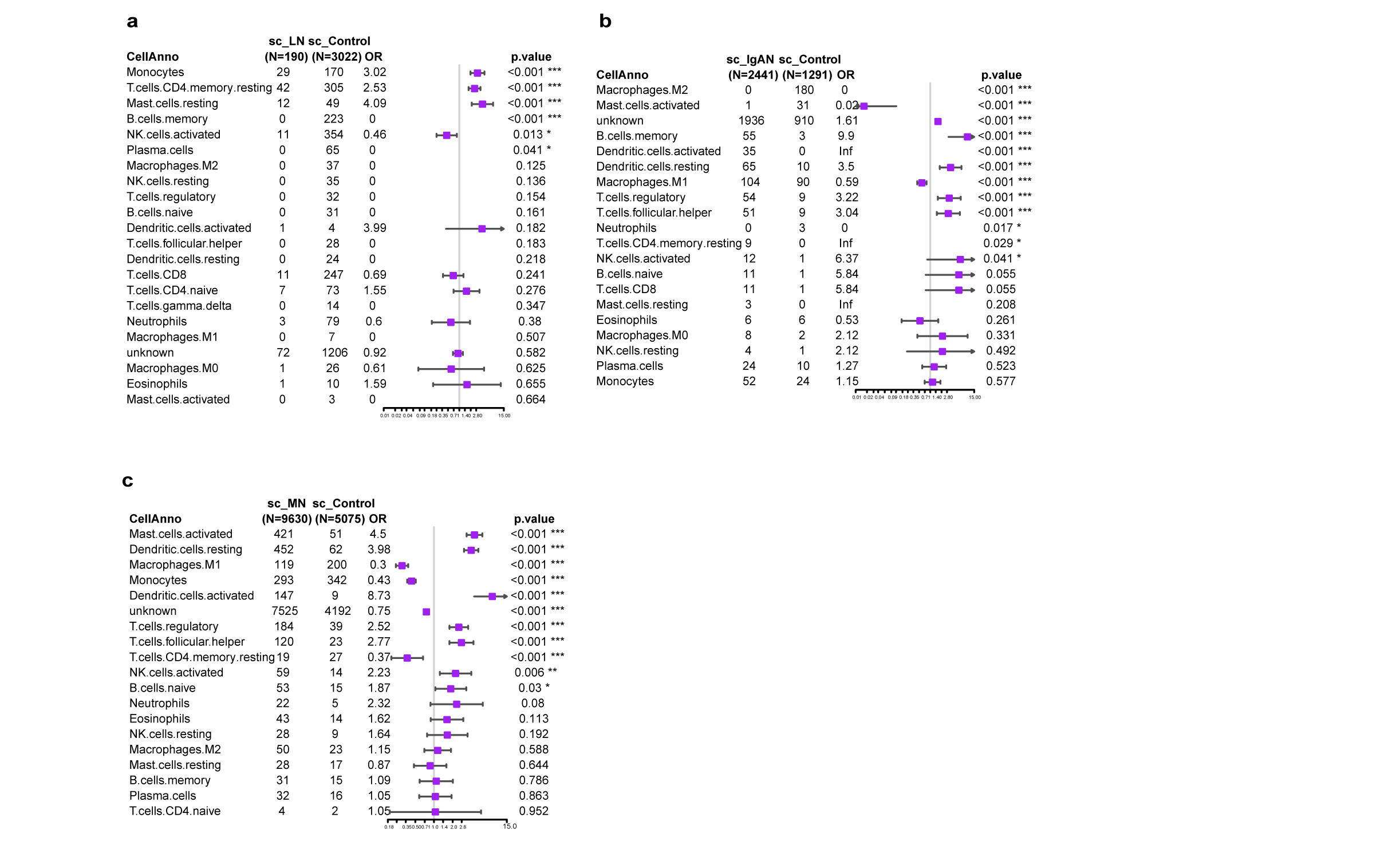


**h**

**g**


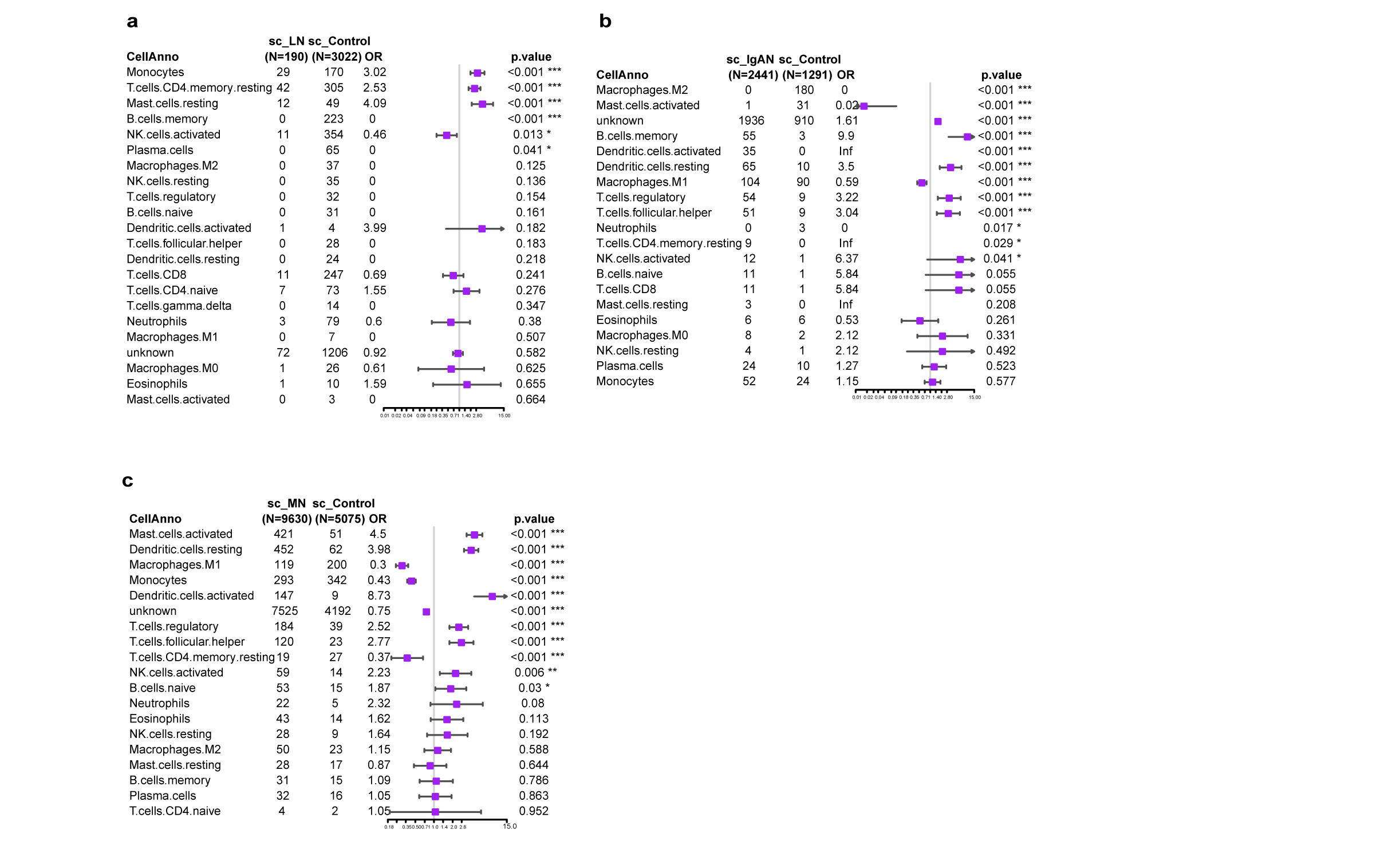

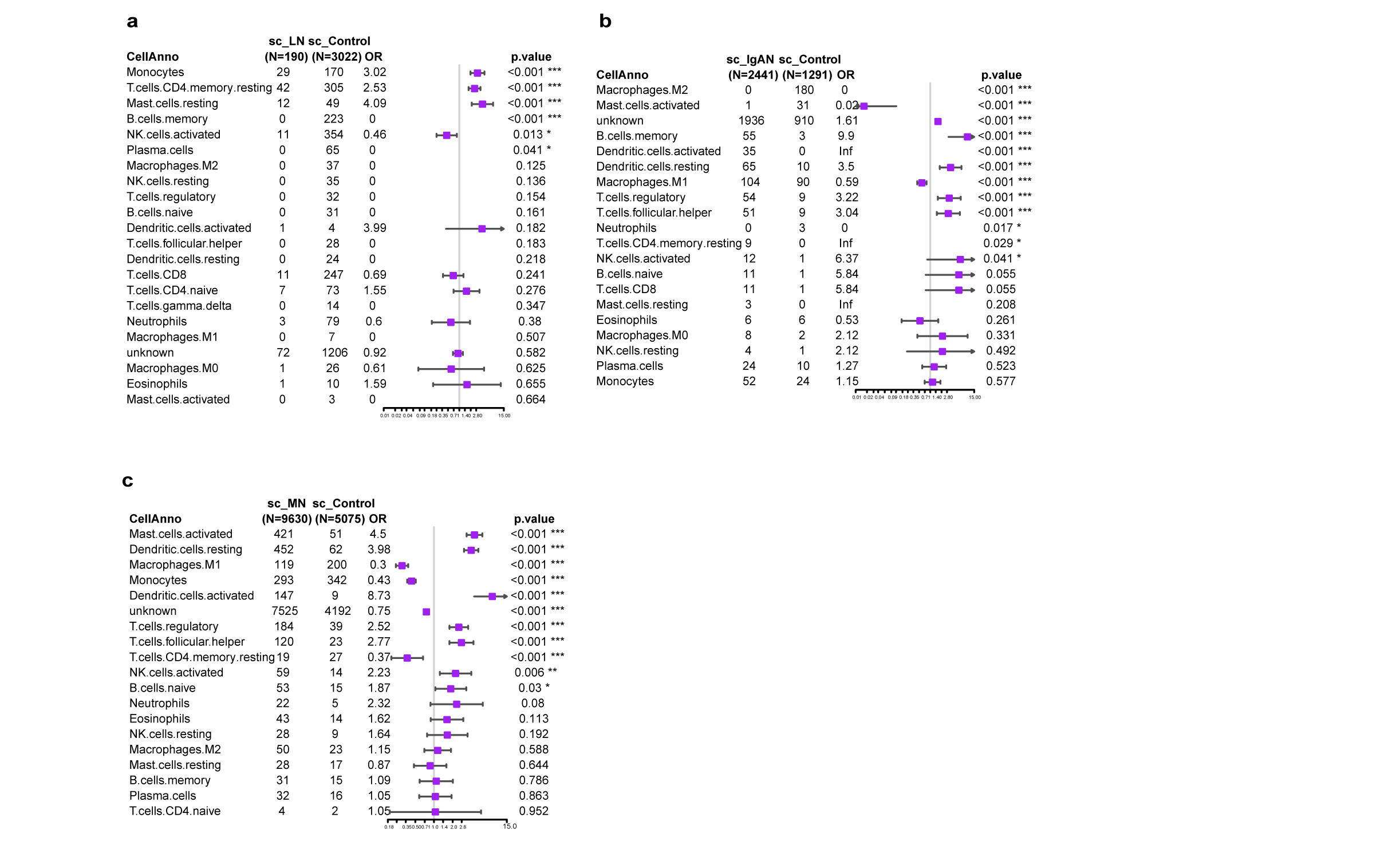

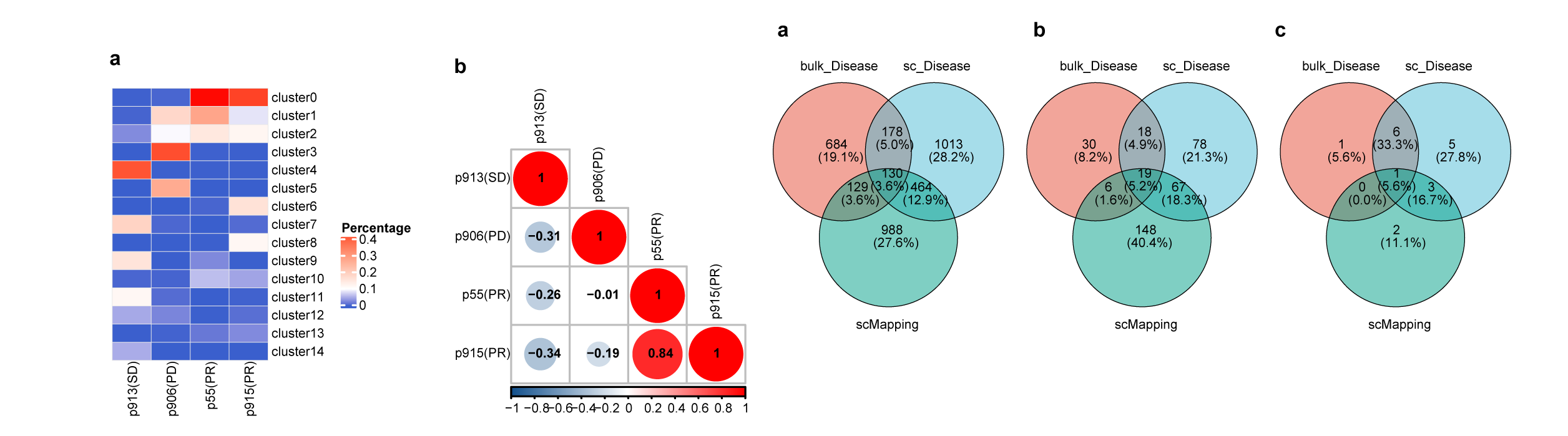


**i**

**j**

**k**

**Supplementary Fig S3. Features difference at the single cell level in nephritis-related diseases.** The overlap between (a) differentially expressed genes (DEGs) and (b) differentially expressed pathways (DEPs) across various types of nephropathy. (c) The overlapping of renal disease-related pathway signatures with those associated with response and prognosis in the IMmotion151 cohort. (d) Chord graph demonstrate representative genes and their corresponding pathways identified in the single-cell RNA sequencing (scRNA) data of kidney-mediated disorders. (e) Violin plot demonstrate that DDX58/IFIH1-mediated induction of interferon-alpha/beta (R-HSA-168928) exhibits higher expression in responders than non-responders from the IMmotion151 dataset. Forest plot of quantitative counting and comparison of 22 different types of immune cells between disease and control groups for the (f) single-cell lupus nephritis (scLN), (g) single-cell IgA nephropathy (scIgAN), and (h) single-cell membranous nephropathy (scMN) datasets. Comparison of (i) genes, (j) pathways and (k) cells identified in bulk RNA data of nephropathy (bulk_Disease), single-cell RNA data of nephropathy (sc_Disease), and bulk-scRNA mapping of ICBs (IMmotion151 mapped to Bi.et al, scMapping).


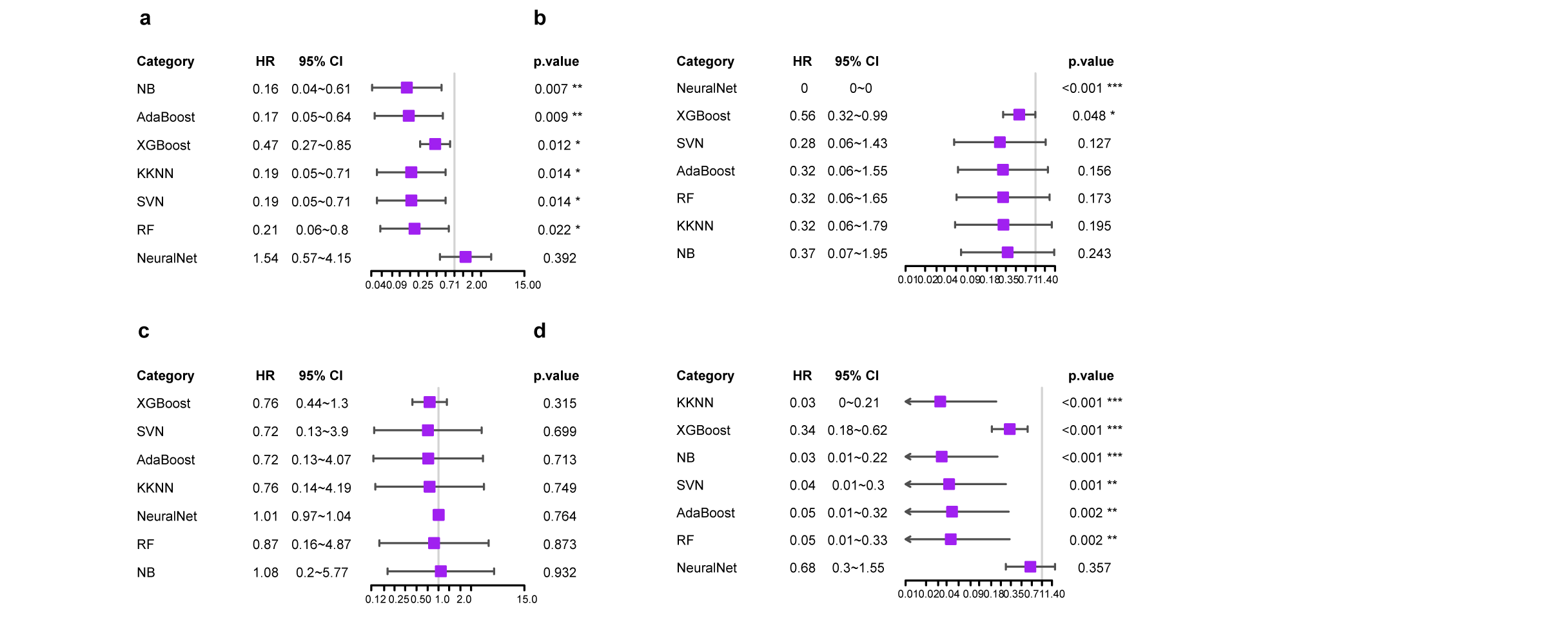


**a**

**b**


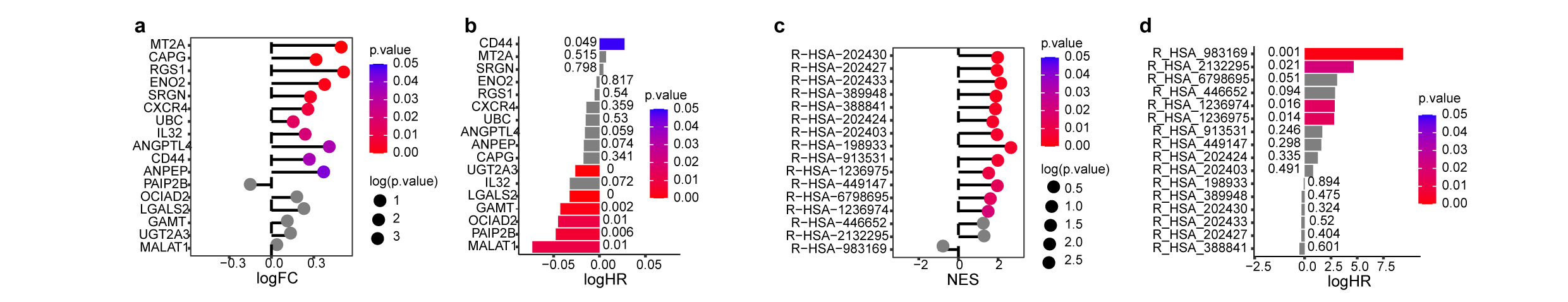

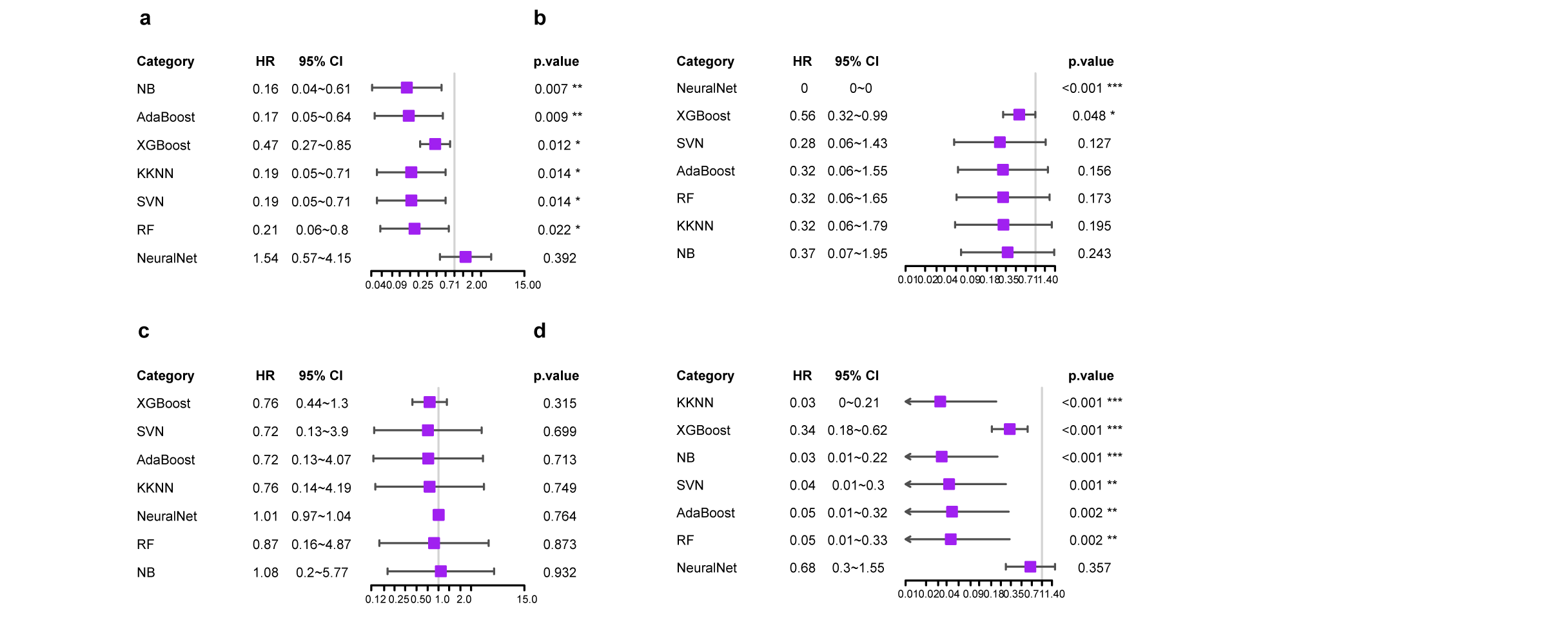

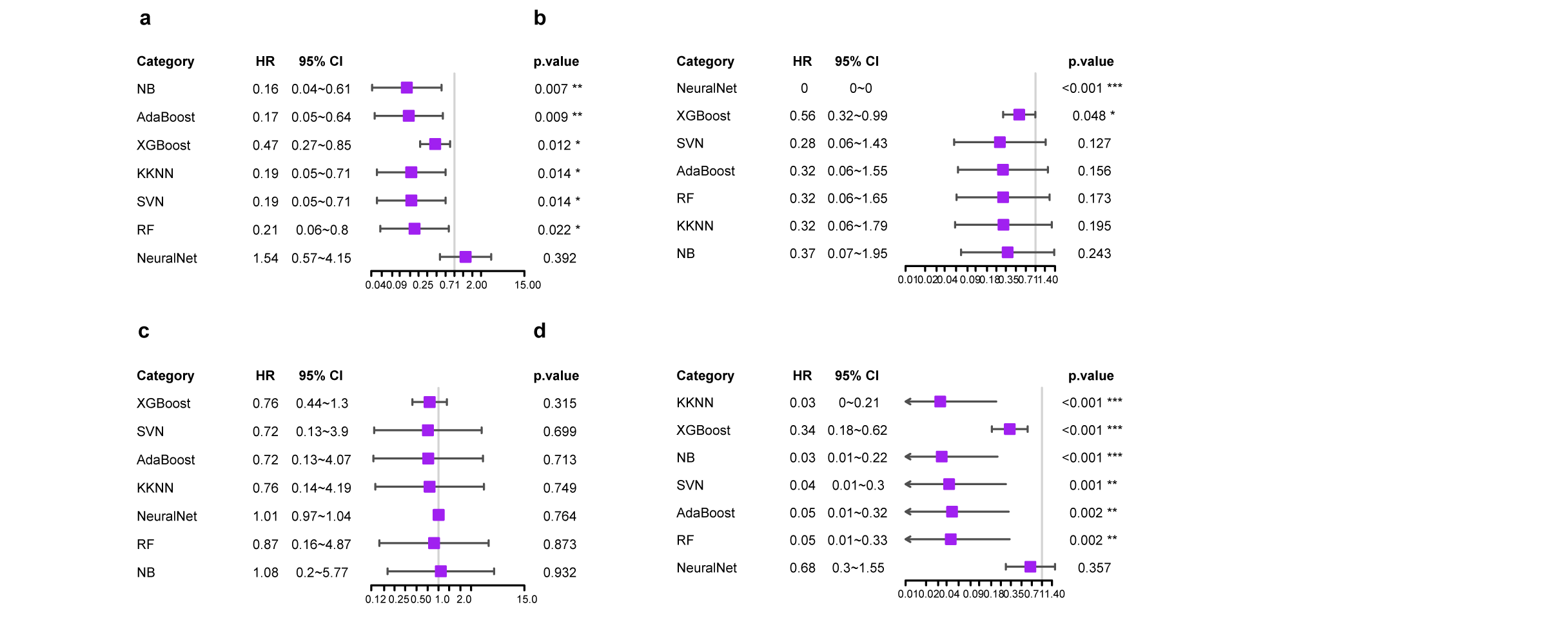

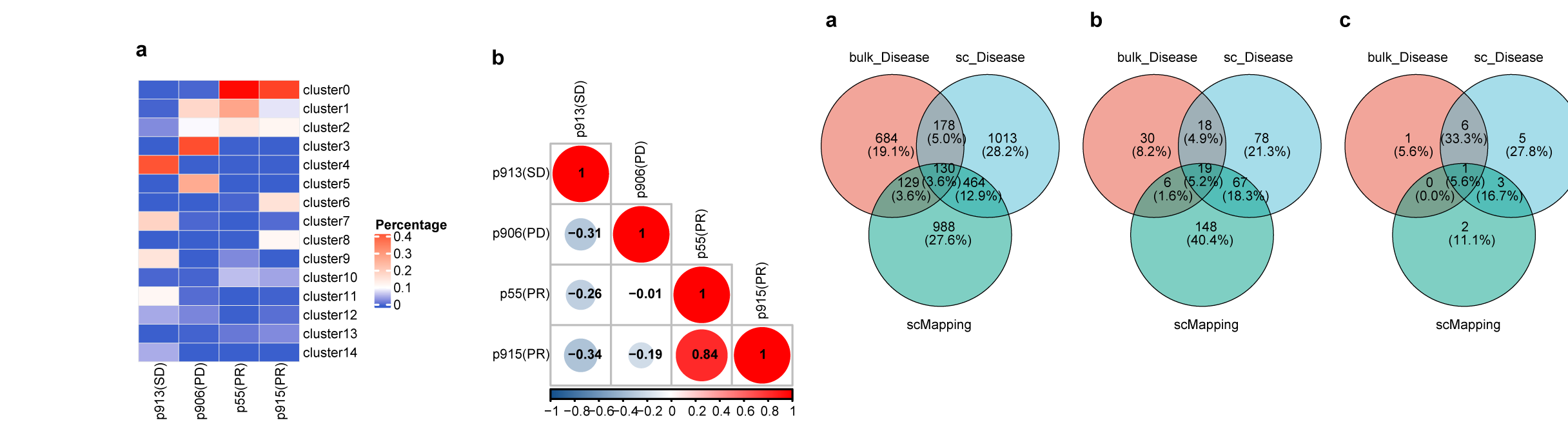

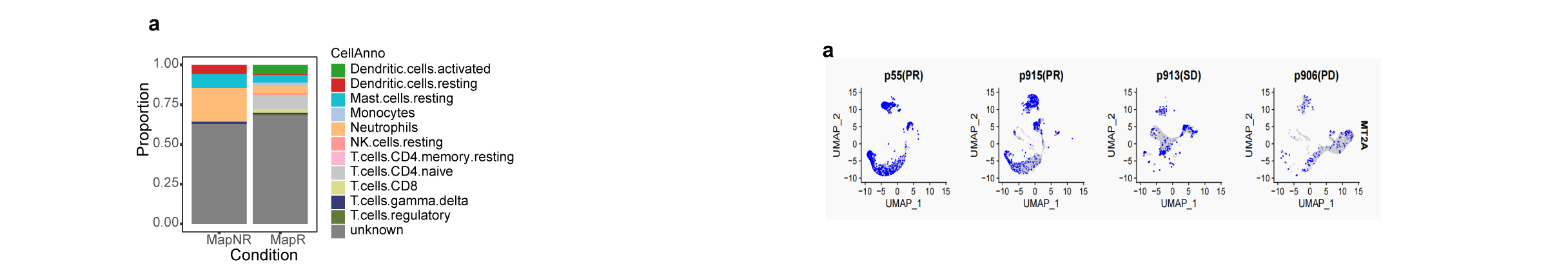


**c**


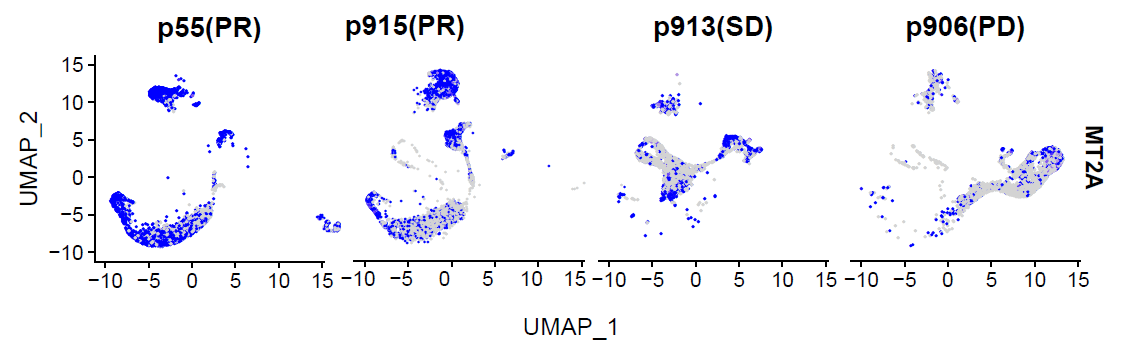


**d**


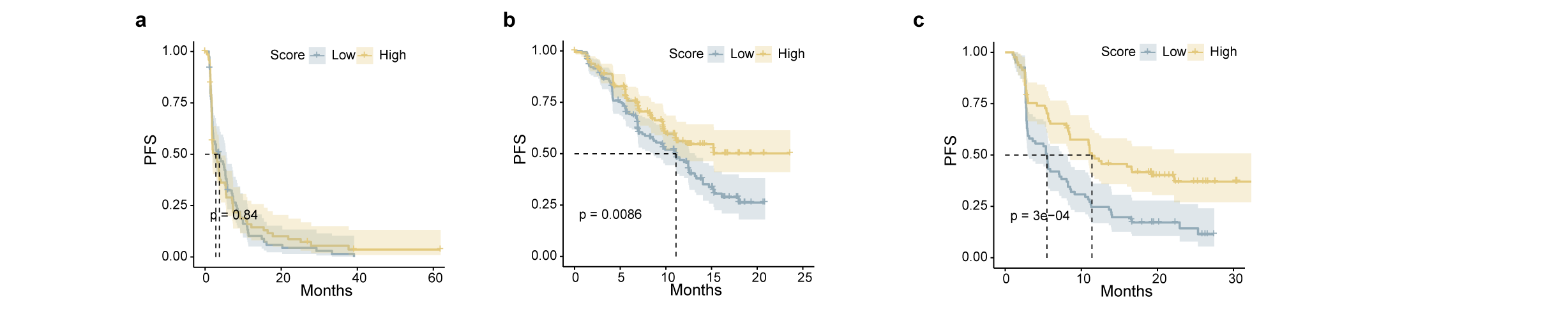

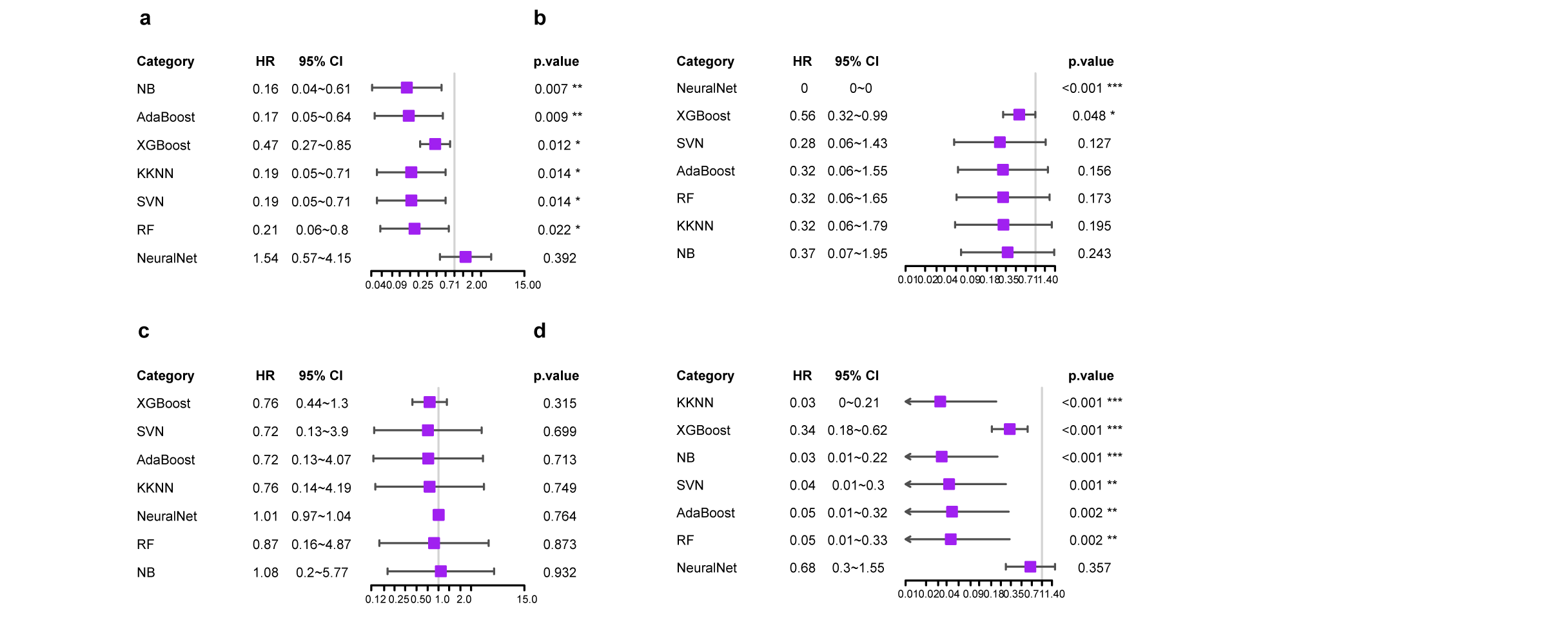


**e**

**f**

**g**

**h**

**i**

**j**

**k**

**n**

**m**

**l**

**o**


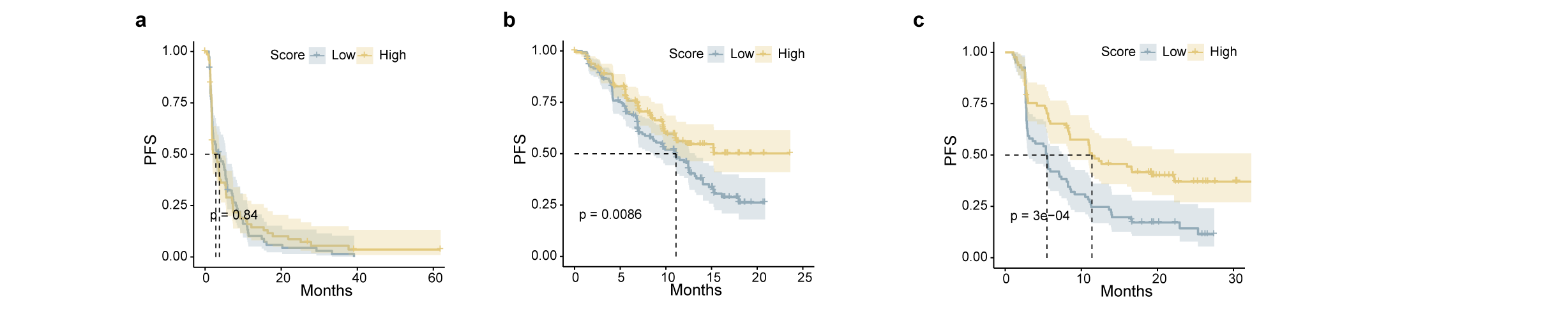


**Supplementary Fig S4. The value of inflammatory signatures to predict immunotherapy response.** (a) Correlations among different patients and cell clusters in Bi et al.. (b) Correlations within different patients in Bi et al.. (c) Differences immune cells (DICs) between mapped effective and ineffective cells in Bi et al. (d) MT2A is upregulated in responders compared to non-responders in Bi et al. Stacked bar chart of immune cells of bulk mapped Bi et al. cells. The lollipop plots illustrate the differential expression of mapping-derived (e) genes and (g) pathways between responders and non-responders in the IMmotion151 cohort. Postive logFC and NES represent up-regulated in responders group. The bar graphs demonstrate predictions for progression-free survival (PFS) using (f) genes and (h) pathways derived from mapping in the IMmotion151 cohort. Negative logHR present the signature was the good prognosis indicator. Prognostic comparison of 7 machine learning models among different datasets. The forest plots illustrate the prognosis effect of each model in terms of Progression-Free Survival (PFS) using inflammatory gene signatures in the cohorts of (i) JAVELIN and (j) IMmotion150, as well as pathway signatures in (k) JAVELIN and (l) IMmotion150. Prognostic stratifying of progression-free survival (PFS) in immunological cohorts through the utilization of integrated inflammatory signatures in the XGBoost model. Specifically, Kaplan-Meier curves demonstrate score in (m) CheckMate, (n) JAVELIN and (o) IMmotion150 cohorts.


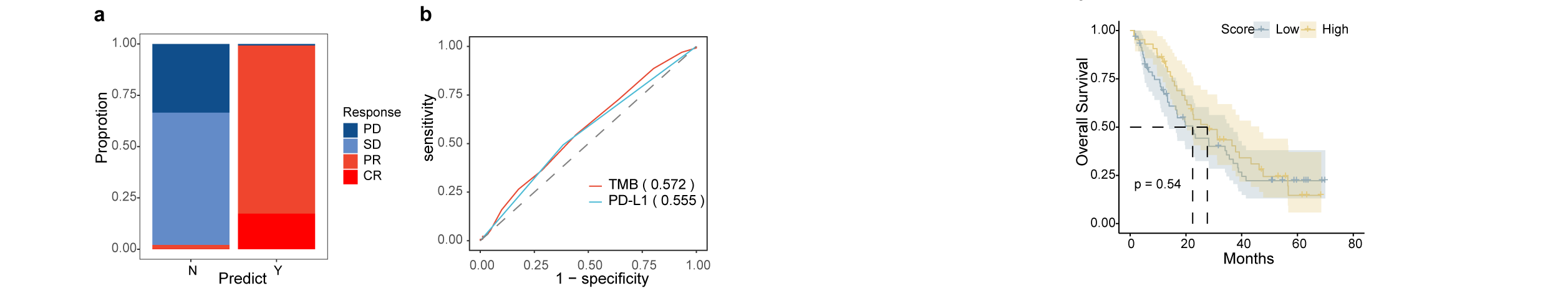
**Supplementary Fig S5. The value of immunogenic signatures for ICB response.** An assessment of the predictive ability of different indicators in the IMmotion151 study. (a) comparison between TIs models predict results and true outcomes, the threshold of models set 0.5. (b) ROC illustrate the response prediction efficacy of two common ICBs indicators that tumor mutational burden (TMB) and programmed death-ligand 1 (PD-L1). (c) Concomitant occurrence among frequent genes mutation. Only 6 pair of genes were co-occurrence significantly. Prognostic comparison of 7 machine learning models among different datasets in single-mutation model. The forest plots illustrate the prognosis effect of each model in terms of Progression-Free Survival (PFS) using immunogenic gene signatures which single mutation models in (d) CheckMate and (e) JAVELIN. Prognostic comparison of 7 machine learning models among different datasets in duo-mutation model. The forest maps in (f) and (g) represent the performance of two different models, CheckMate and JAVELIN, respectively, when combined immunogenic signatures were used as input for prediction.


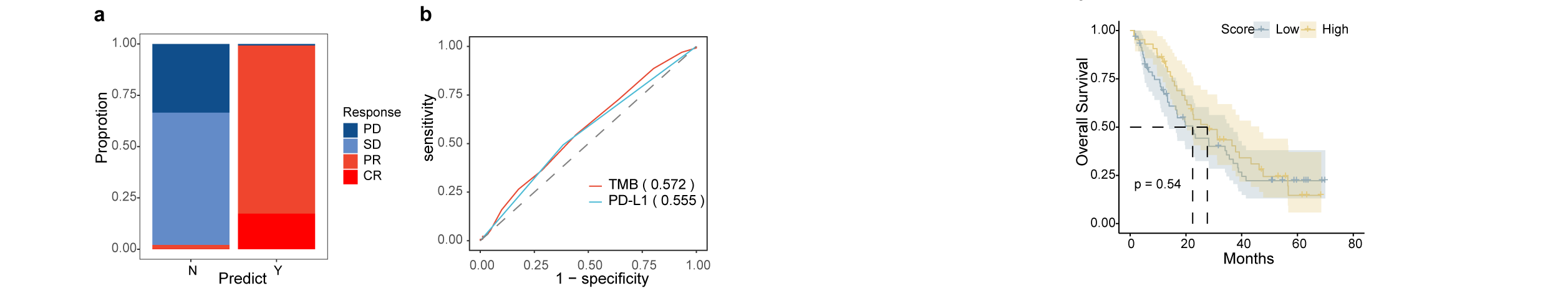

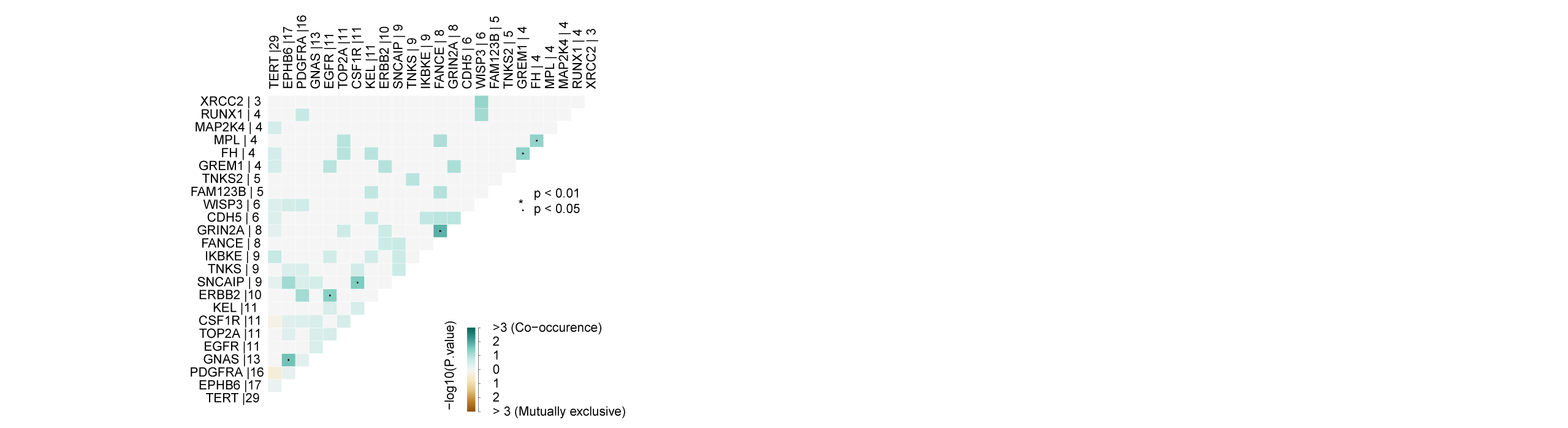

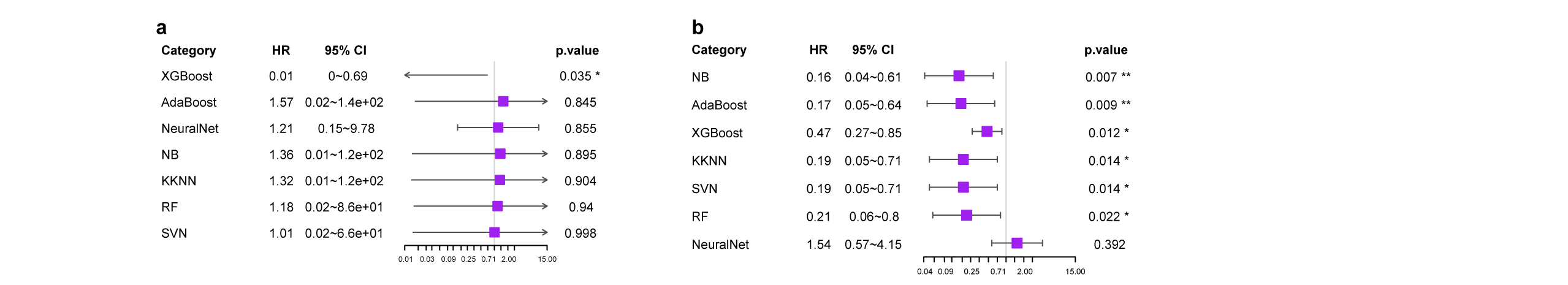

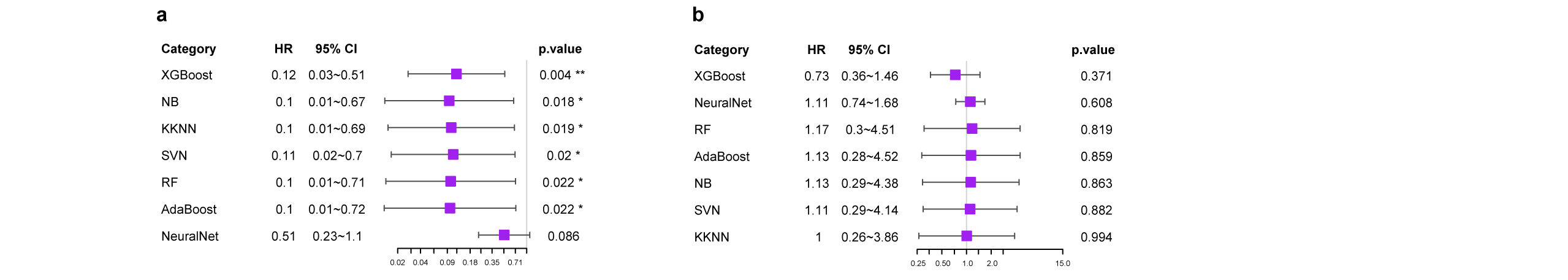


**e**

**f**

**g**

**c**

**d**


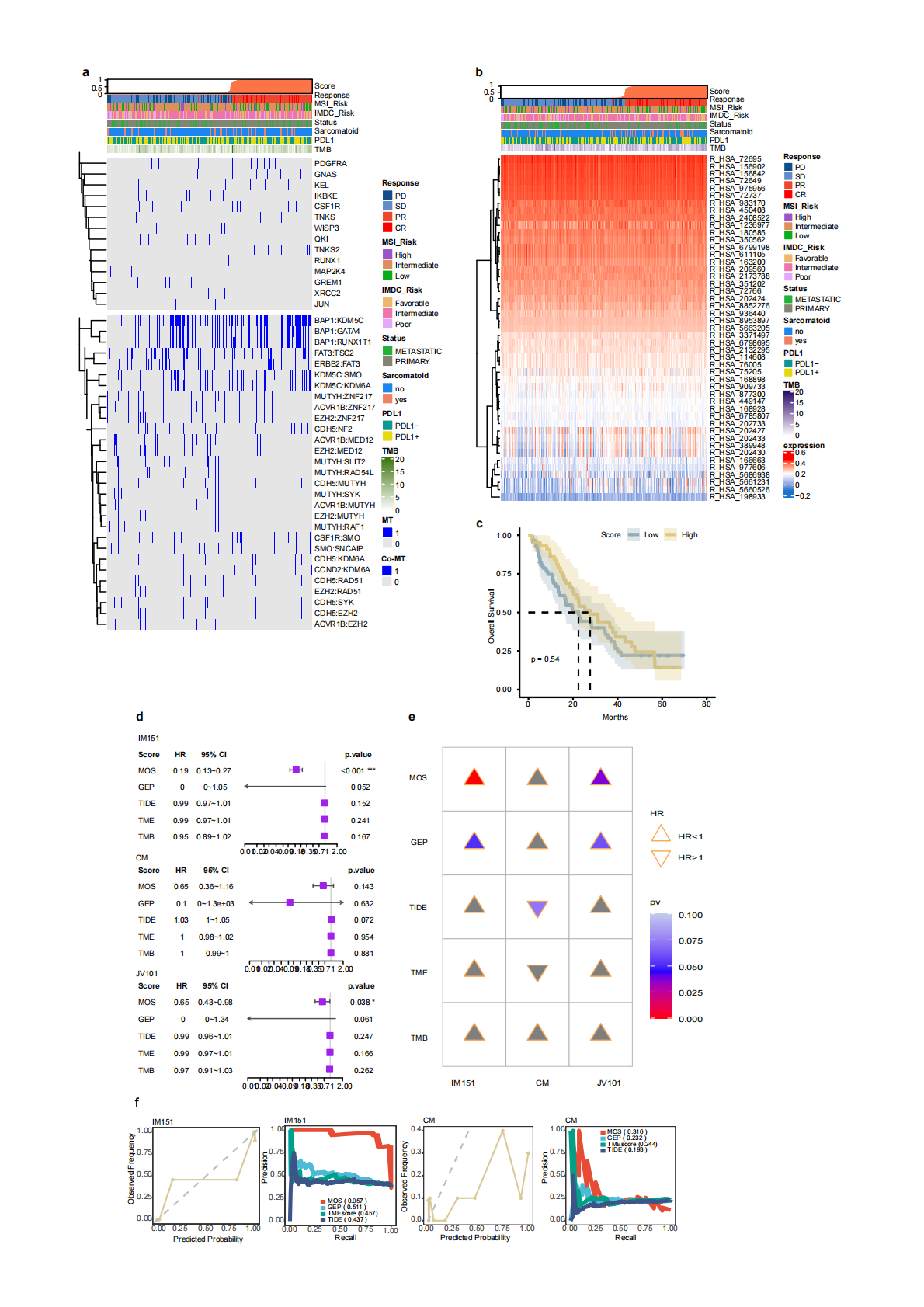


**Supplementary Fig S6. The value of TIs for ICB effectiveness prediction.** The landscape of the model's input pathway and mutation signatures in the IMmotion151 cohort. Kaplan-Meier curves for TIs model score in CheckMate. The heatmaps illustrate the following: (a) the mutation profiles corresponding to the mutation signatures, and (b) the expression patterns of pathway signatures. (c) Kaplan-Meier curves for TIs model score in CheckMate. Scores could not be stratifying overall survival (OS) significantly in CheckMate. A comparative analysis between our multi-omics model (TIs) and other immune prediction approaches in prognostic efficacy of progression-free survival (PFS) within the IMmotion151 (IM151), CheckMate (CM), and JAVELIN (JV101) cohorts. The results are visually presented through (d) forest plots and (e) heatmap. (f) precision-recall (PR) curves and calibration plots of IM151 and CM.

**Supplementary Fig S7. Correlation of TIs risk score with different characteristics.** The correlations between the model score and various clinical variables within the IMmotion151 and CheckMate cohorts. The correlations are analyzed separately for each cohort. In IMmotion151, the correlations between the model score and three clinical variables are examined: (a) IMDC risk score, (b) MSKCC score, and (c) metastatic status. Similarly, in the CheckMate cohort, the correlations between the model score and three clinical variables are studied: (d) IMDC risk score, (e) MSKCC score, and (f) metastatic status. Correlations between TIs model score and published functional features in the IMmotion151. (g) hallmark pathways, (h) TME-related signatures. TIs model score used for stratifying progress free survival (PFS) in subsets with low tumor mutational burden (TMB-LOW, i) and negative Programmed Death-Ligand 1 expression (j).


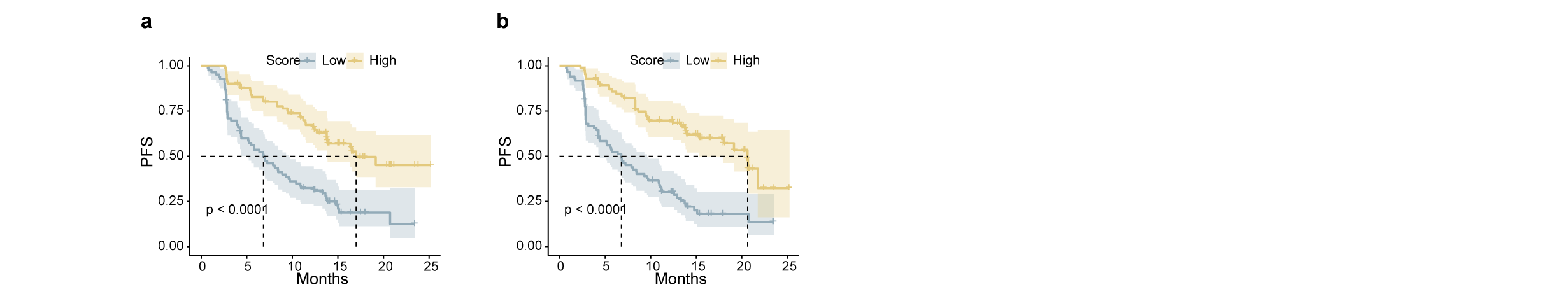

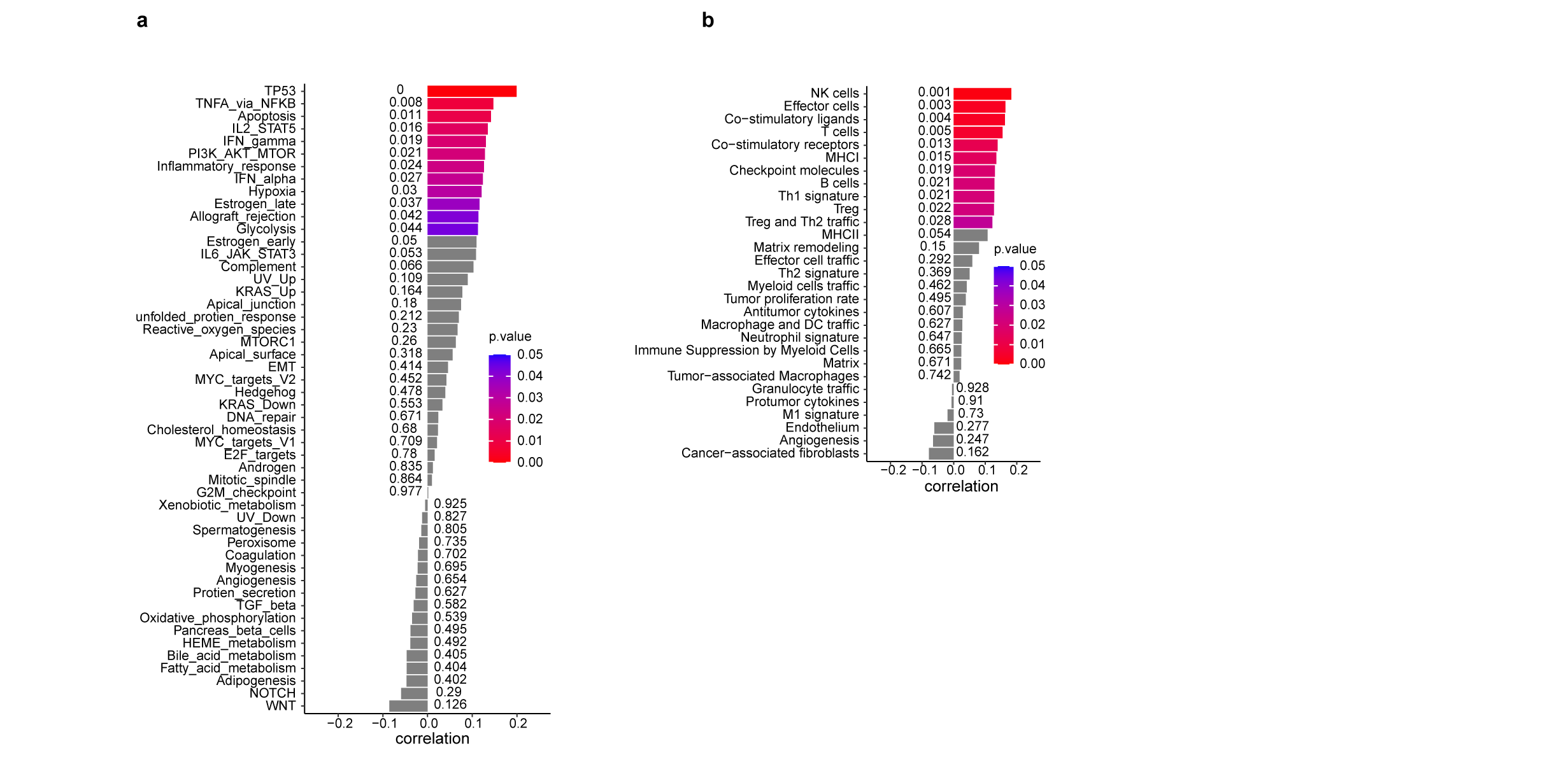

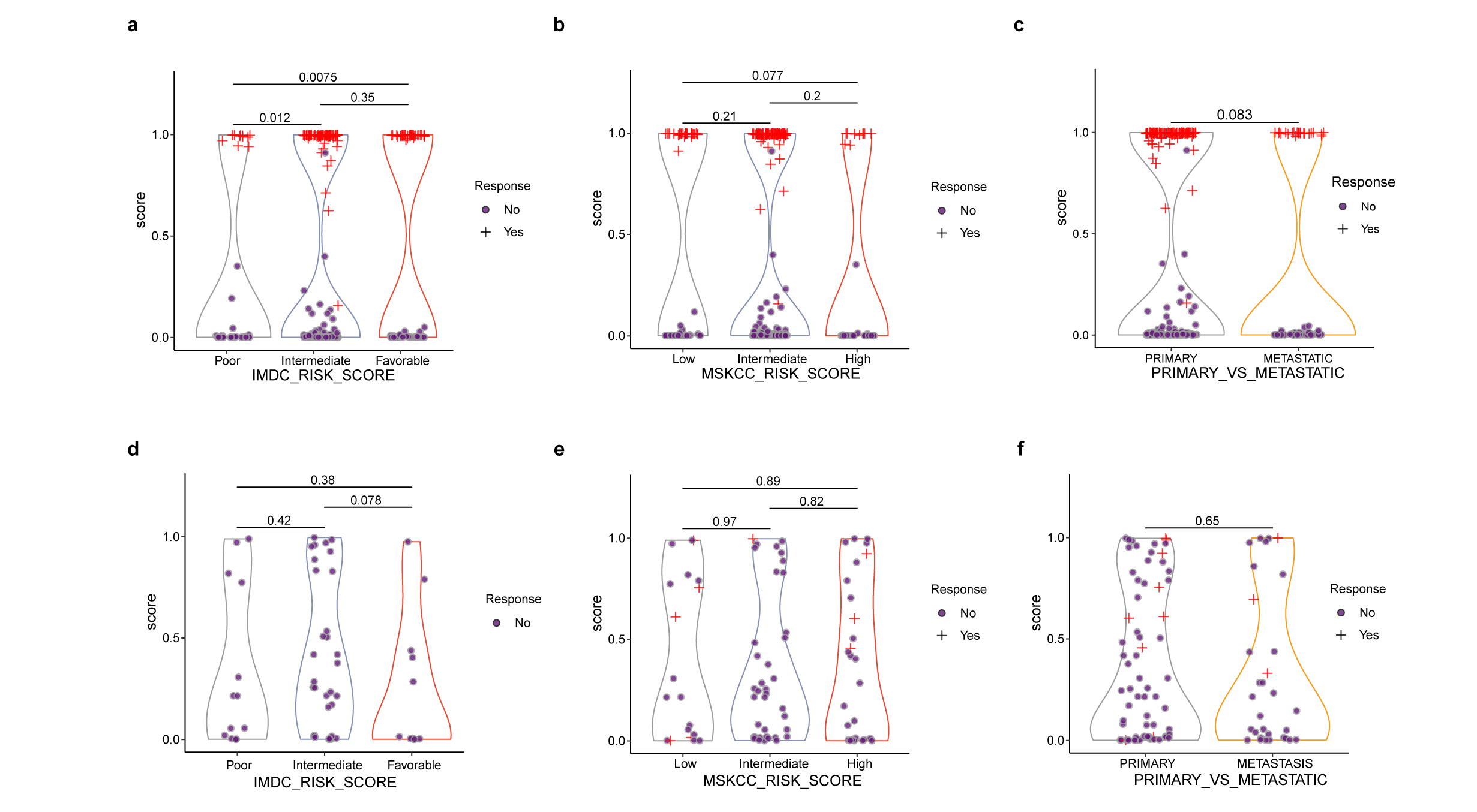

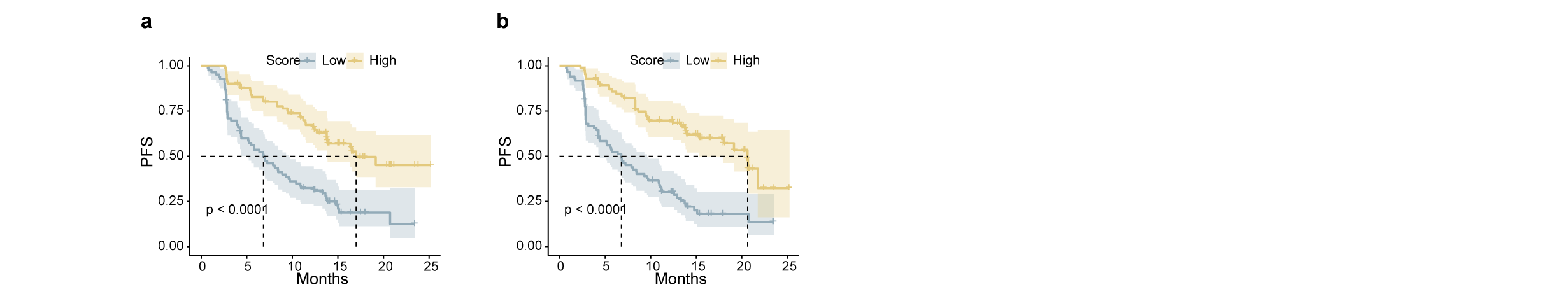

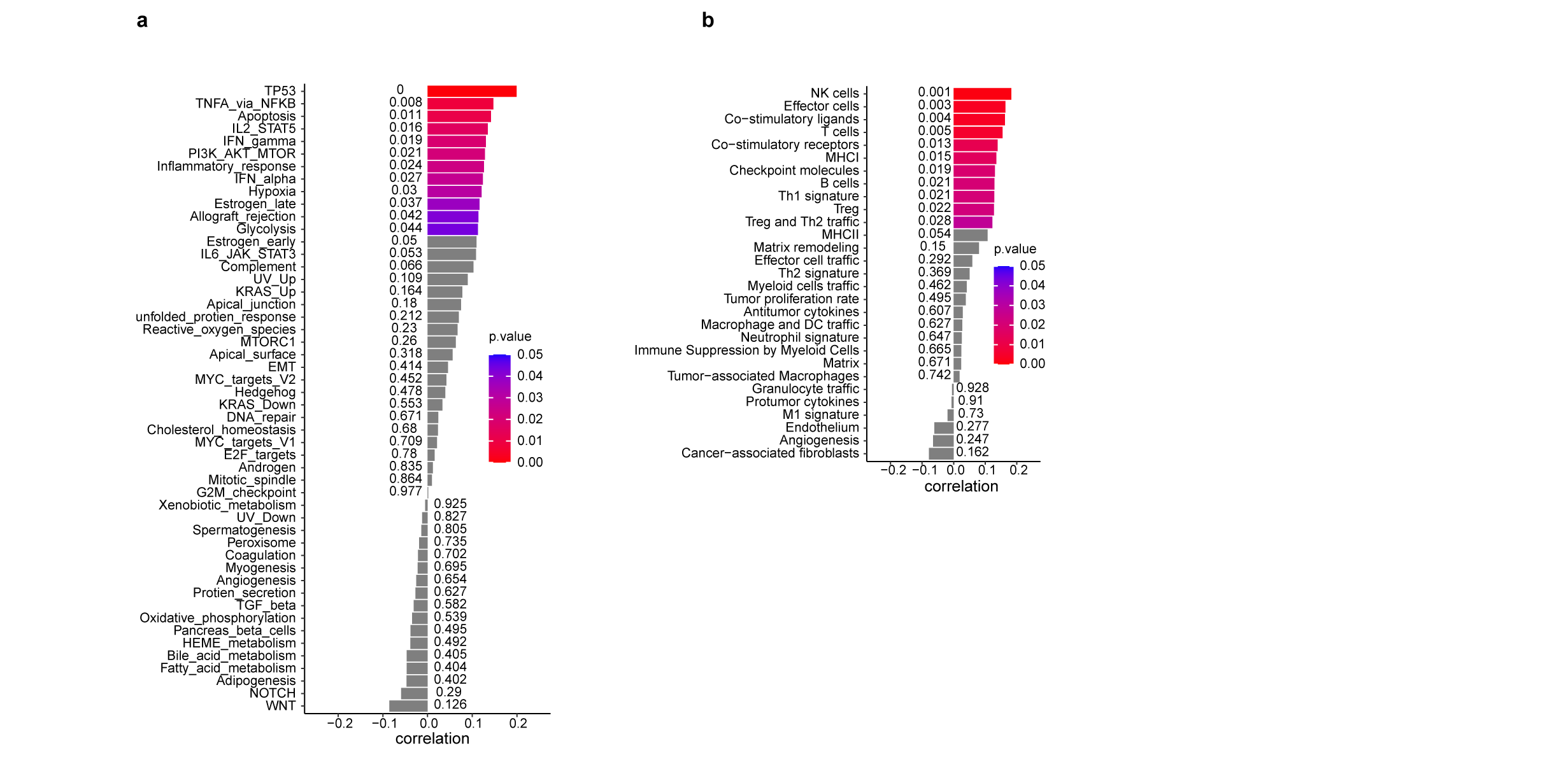


**f**

**a**

**b**

**c**

**d**

**e**

**j**

**i**

**g**

**h**

**k**

**k**


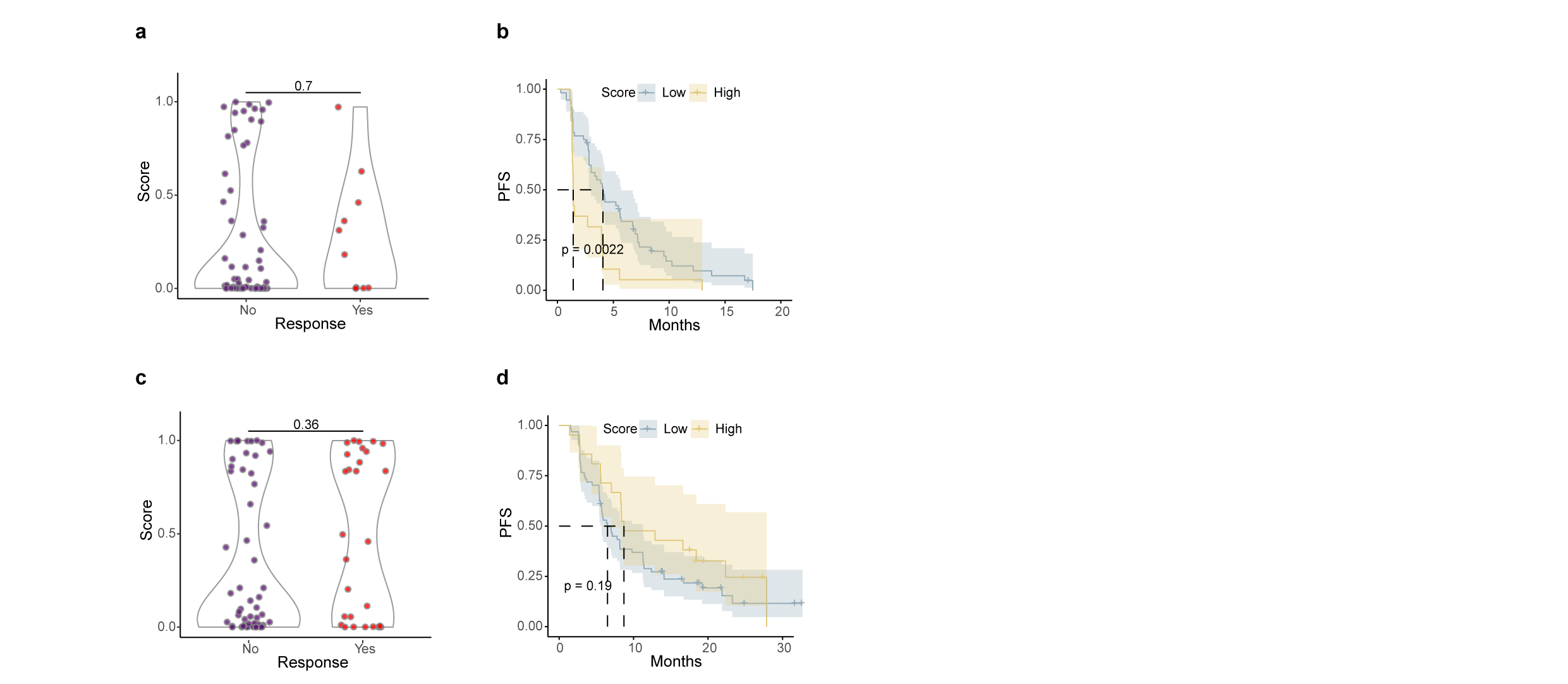


**Supplementary Fig S8. The application of TIs model in predicting response and survival outcomes under various conditions.** Specifically, it showcases the performance of TIs model to (a) predict response and (b) stratifying progression-free survival (PFS) for non-small cell lung cancer (NSCLC) patients undergoing docetaxel treatment in the Poplar cohort. Additionally, bottom figures demonstrates the performance of TIs model to (c) predict response and (d) stratifying PFS for clear cell renal cell carcinoma (ccRCC) patients undergoing sunitinib treatment in the IMmotion150.


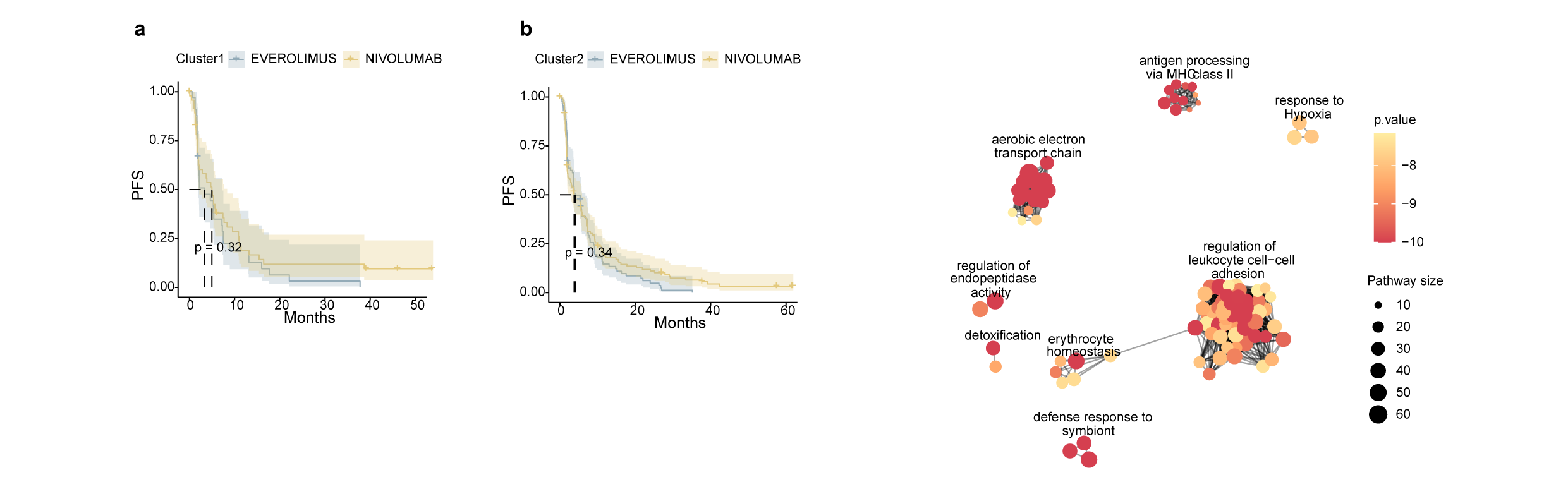

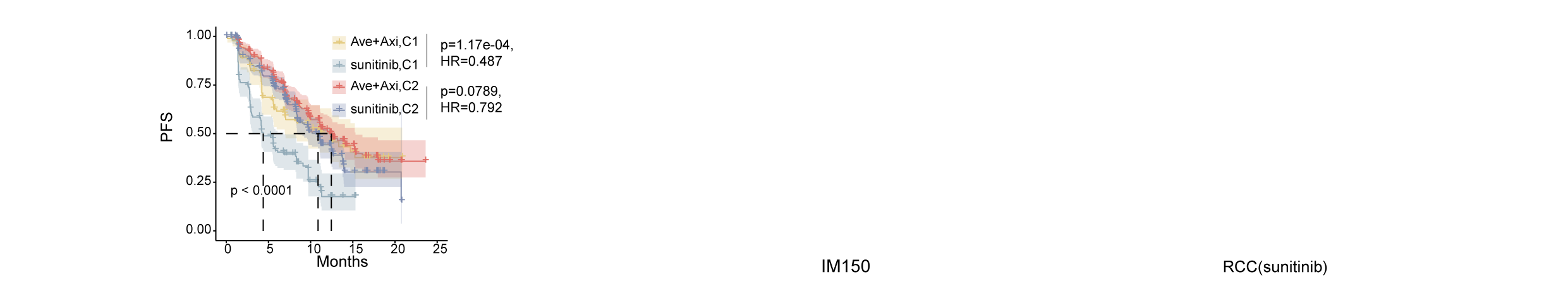

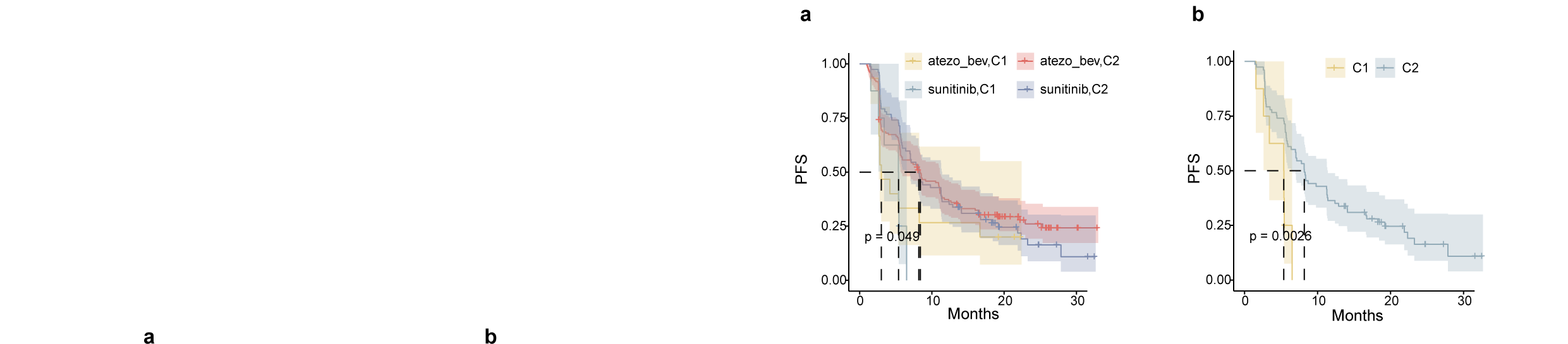

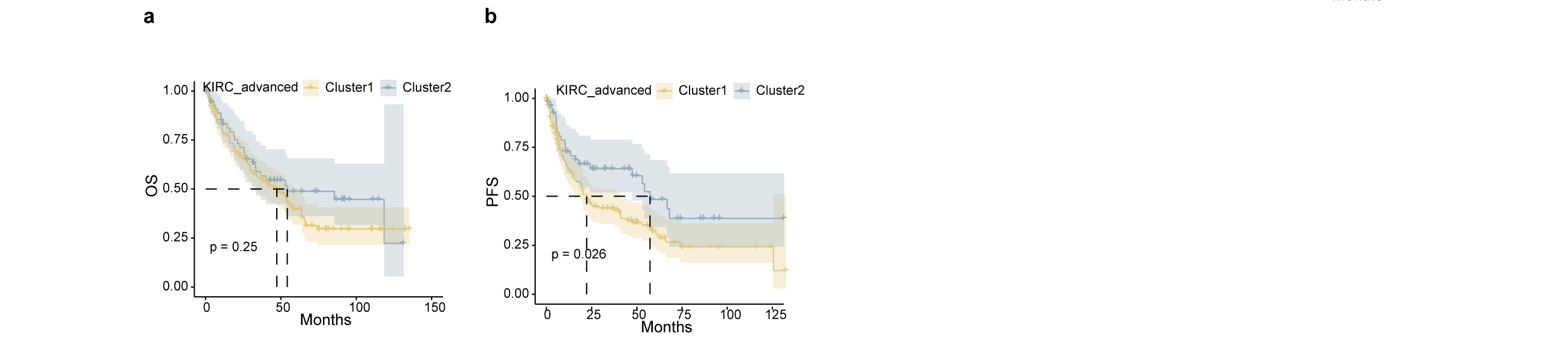

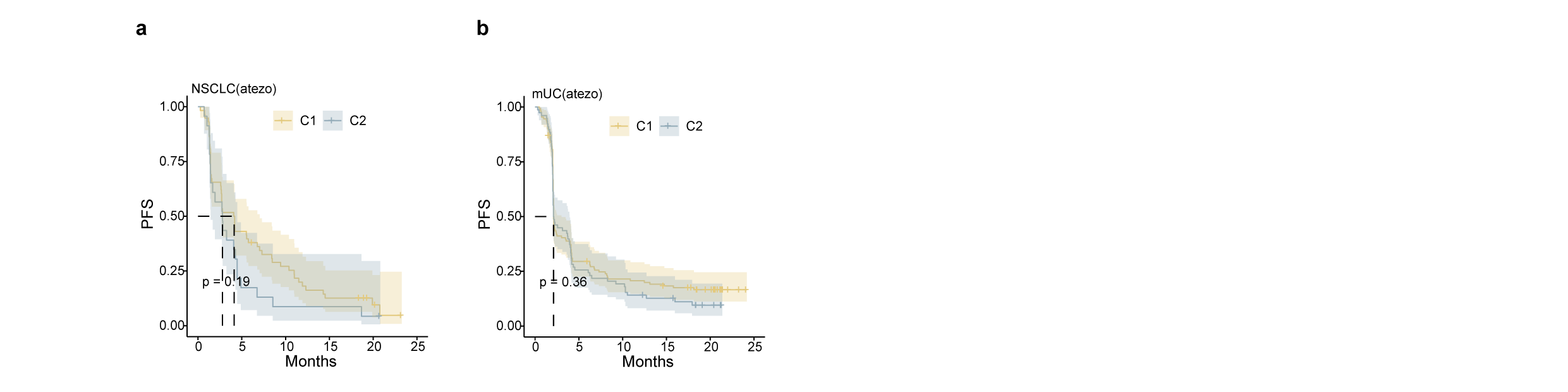


**f**

**a**

**b**

**c**

**d**

**e**

**g**

**h**

**Supplementary Fig S9. Features of TIs was specific for ccRCC patients.** (a) Net graph of pathways cluster that significant enriched by kidney immune disorders derived genes. Clustering results of GO pathways enriched by kidney immune disorders derived genes. (b) Kapan-Meier progress free survival curves of different combinations of treatments and clusters in JAVELIN. Only in cluster1 combined treatment were better than monotherapy in prognosis significantly. (c) presents the survival curves based on a comprehensive condition classification in IMmotion150. Notably, (d) reveals that patients in cluster 2 demonstrate a more favorable prognosis compared to cluster 1 in IMmotion150. The Kaplan-Meier overall survival (OS) and PFS curves for patients with advanced clear cell renal cell carcinoma (ccRCC) in the TCGA cohort, grouped based on different clusters. (e) presents the overall survival (OS) curves, while (f) displays the progression-free survival (PFS) curves. Also cluster2 were the good indicators in TCGA ccRCC significantly. Kidney immune disorder derived clusters were not correlated with different prognosis in non-ccRCC patients received ICBs treatment. Kaplan-Meier PFS curves for (g) IMvigor210, which includes patients with metastatic urothelial carcinoma (mUC), and (h) Poplar, which comprises patients with non-small cell lung cancer (NSCLC).
