# Supplementary figures and images for: A multi-omics features-based approach integrating immunogenicity and inflammation enhances immunotherapy benefit in clear cell renal cell carcinoma
Source: Front Cell Dev Biol. 2026 Jan 20;13:1596719. doi: 10.3389/fcell.2025.1596719 (PMC12864441; doi:10.3389/fcell.2025.1596719)

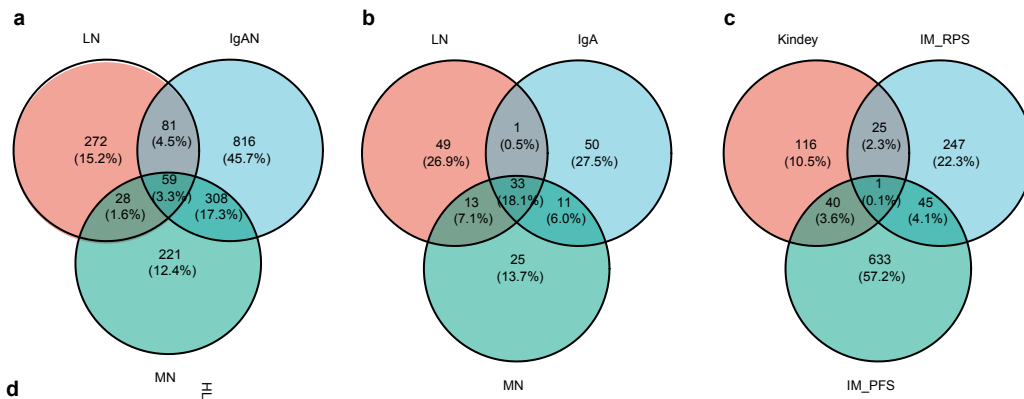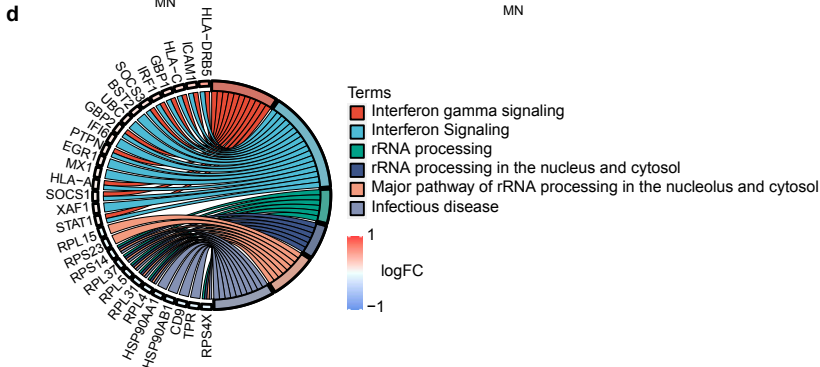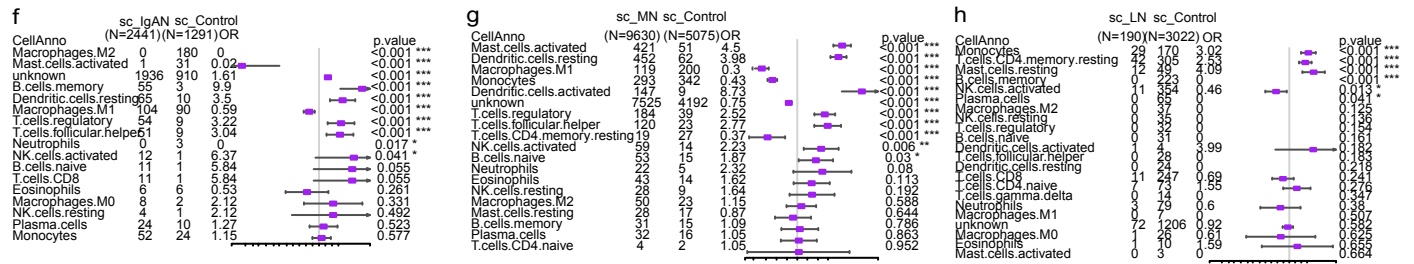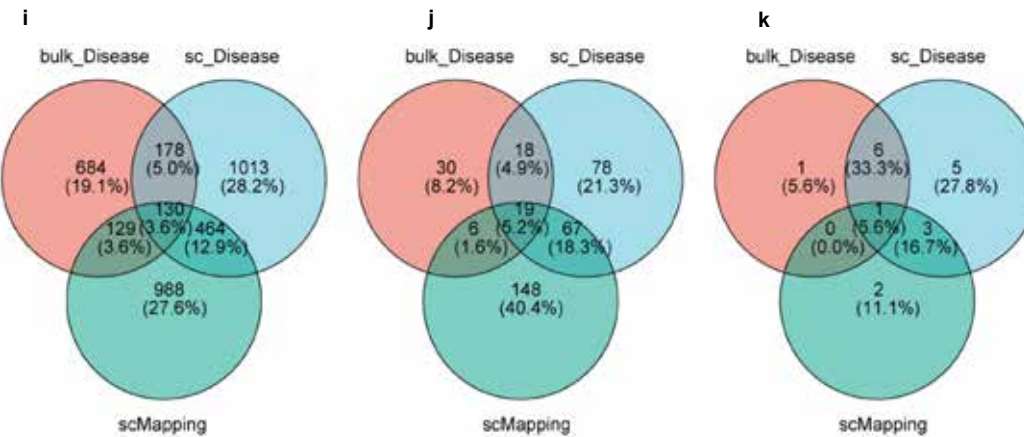

Supplement: Supplementary file 1 [file Presentation1.zip › Supplementary material presentation/Fig S3.pdf]

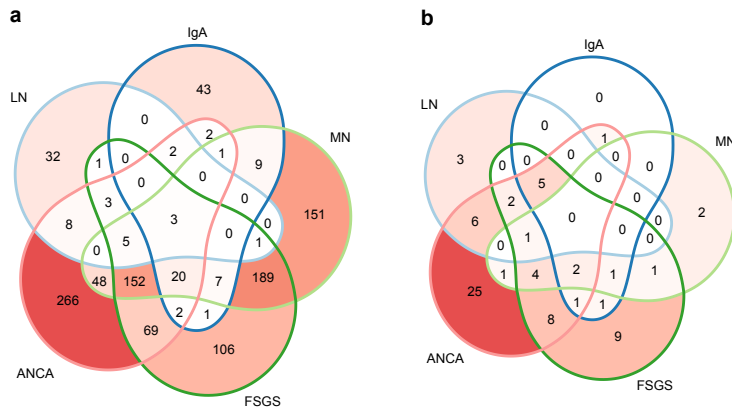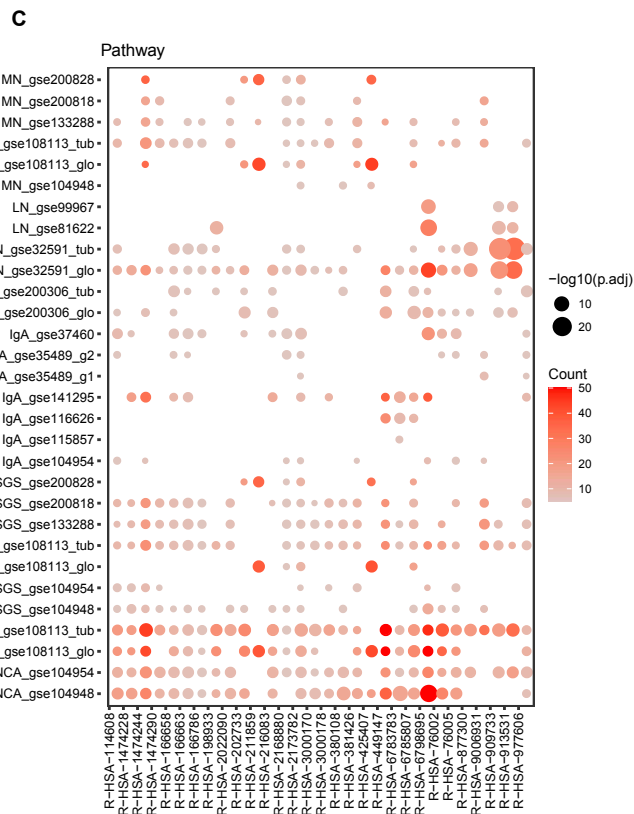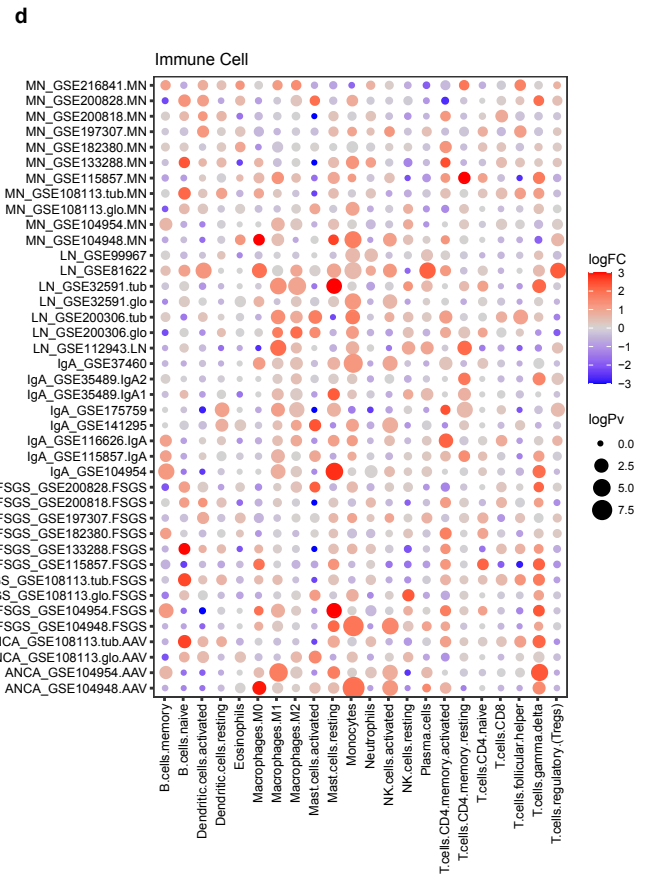

Supplement: Supplementary file 1 [file Presentation1.zip › Supplementary material presentation/Fig S2.pdf]

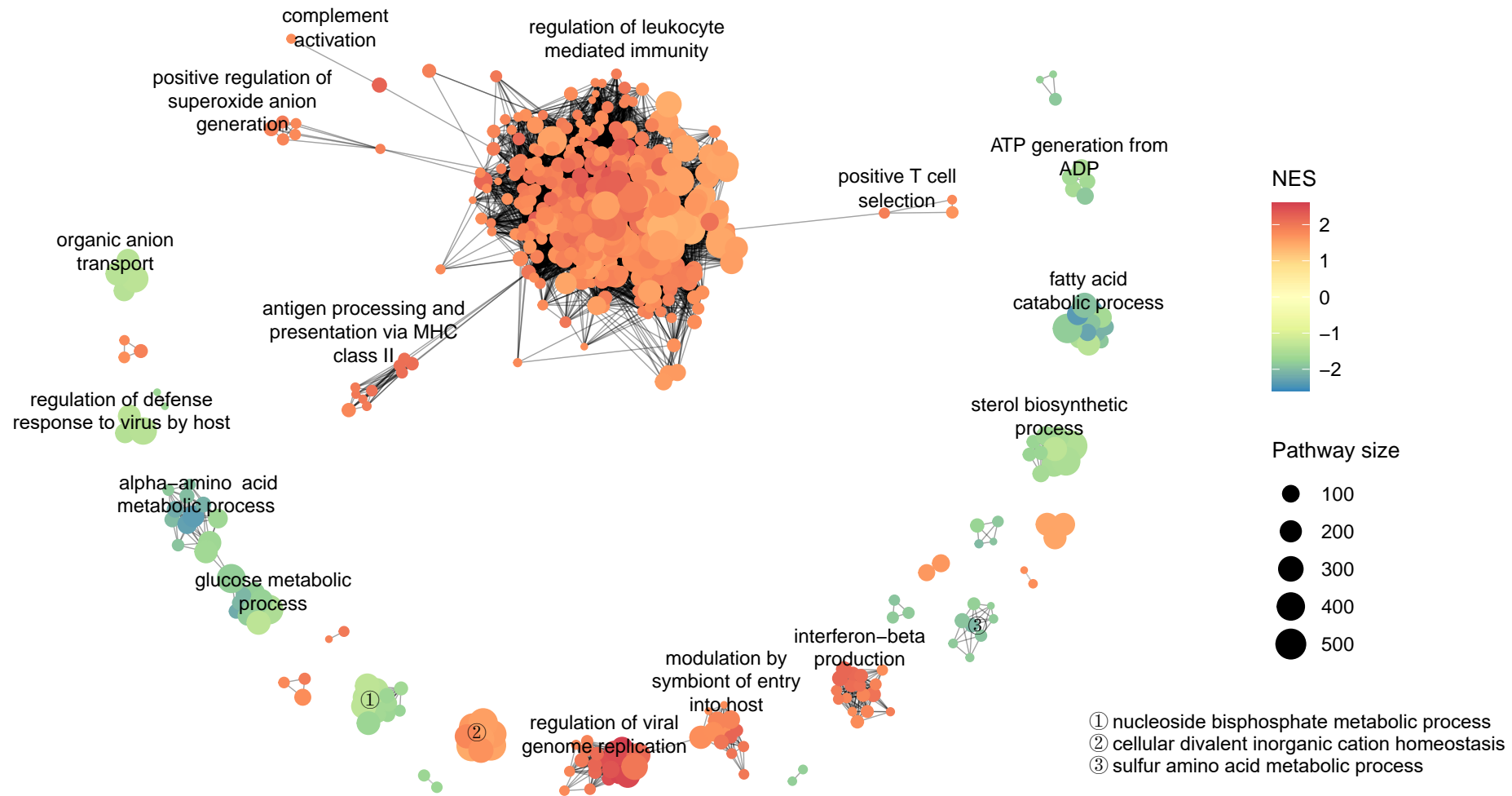

Supplement: Supplementary file 1 [file Presentation1.zip › Supplementary material presentation/Fig S1.pdf]

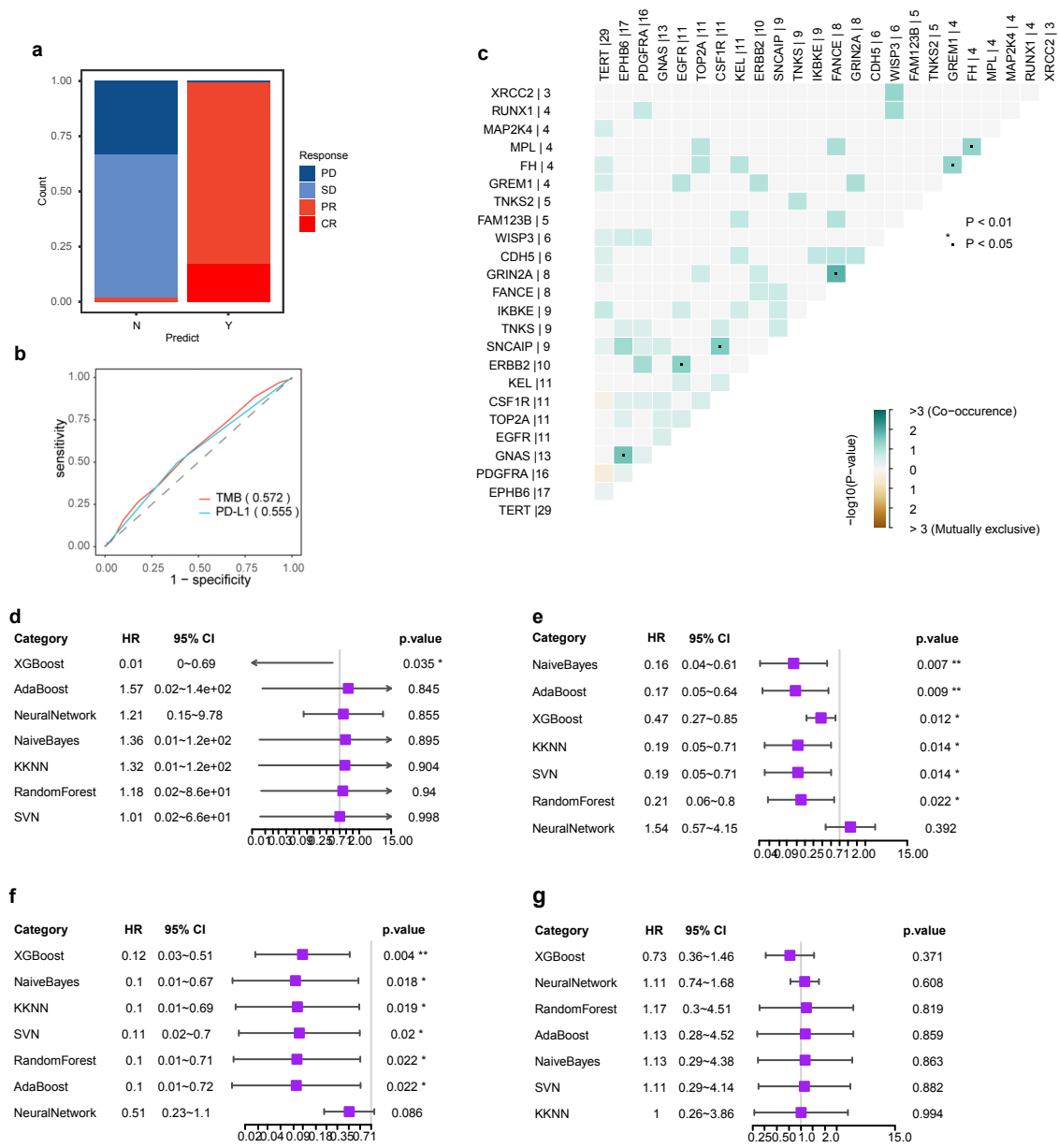

Supplement: Supplementary file 1 [file Presentation1.zip › Supplementary material presentation/Fig S5.pdf]

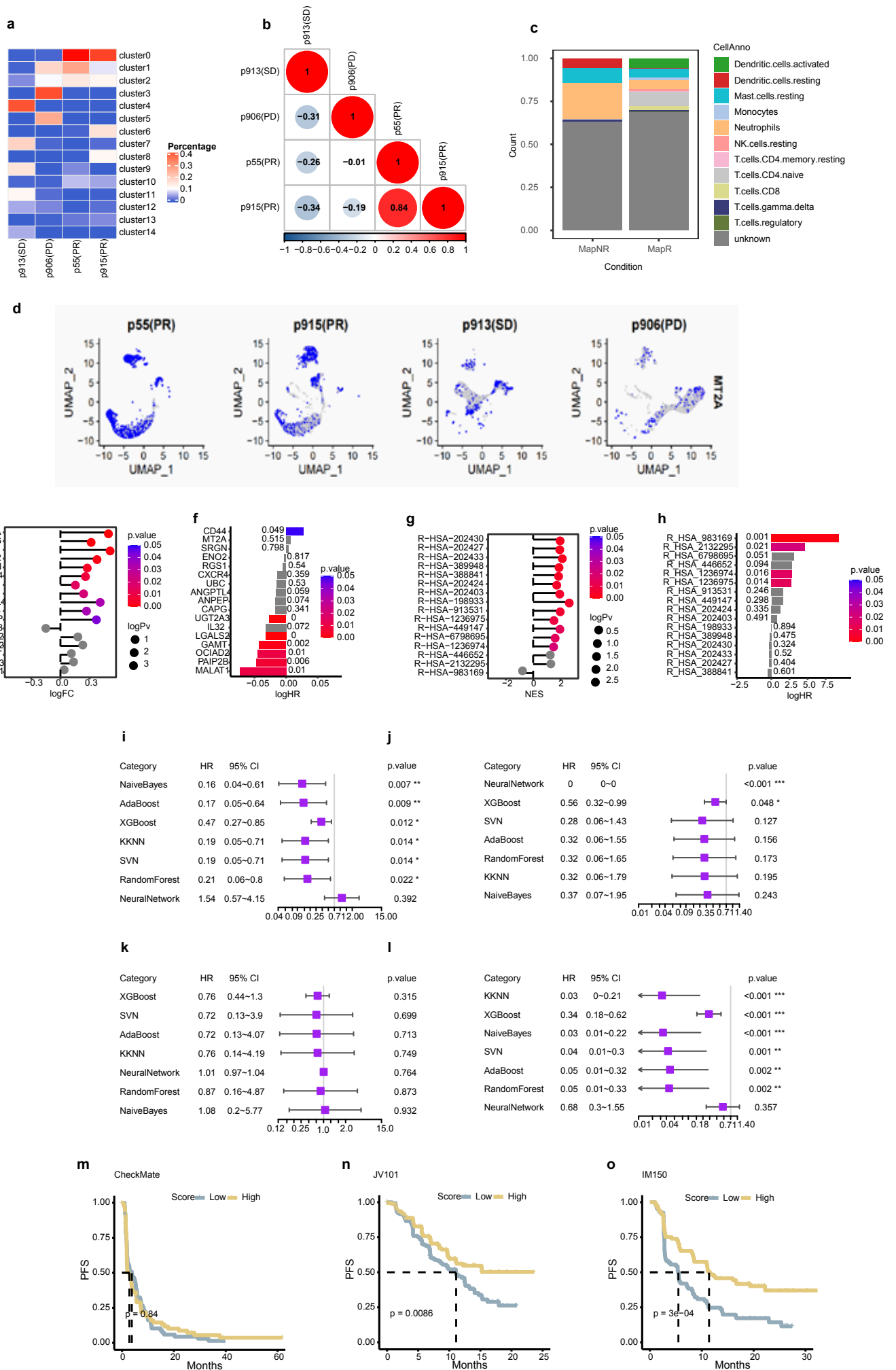

Supplement: Supplementary file 1 [file Presentation1.zip › Supplementary material presentation/Fig S4.pdf]

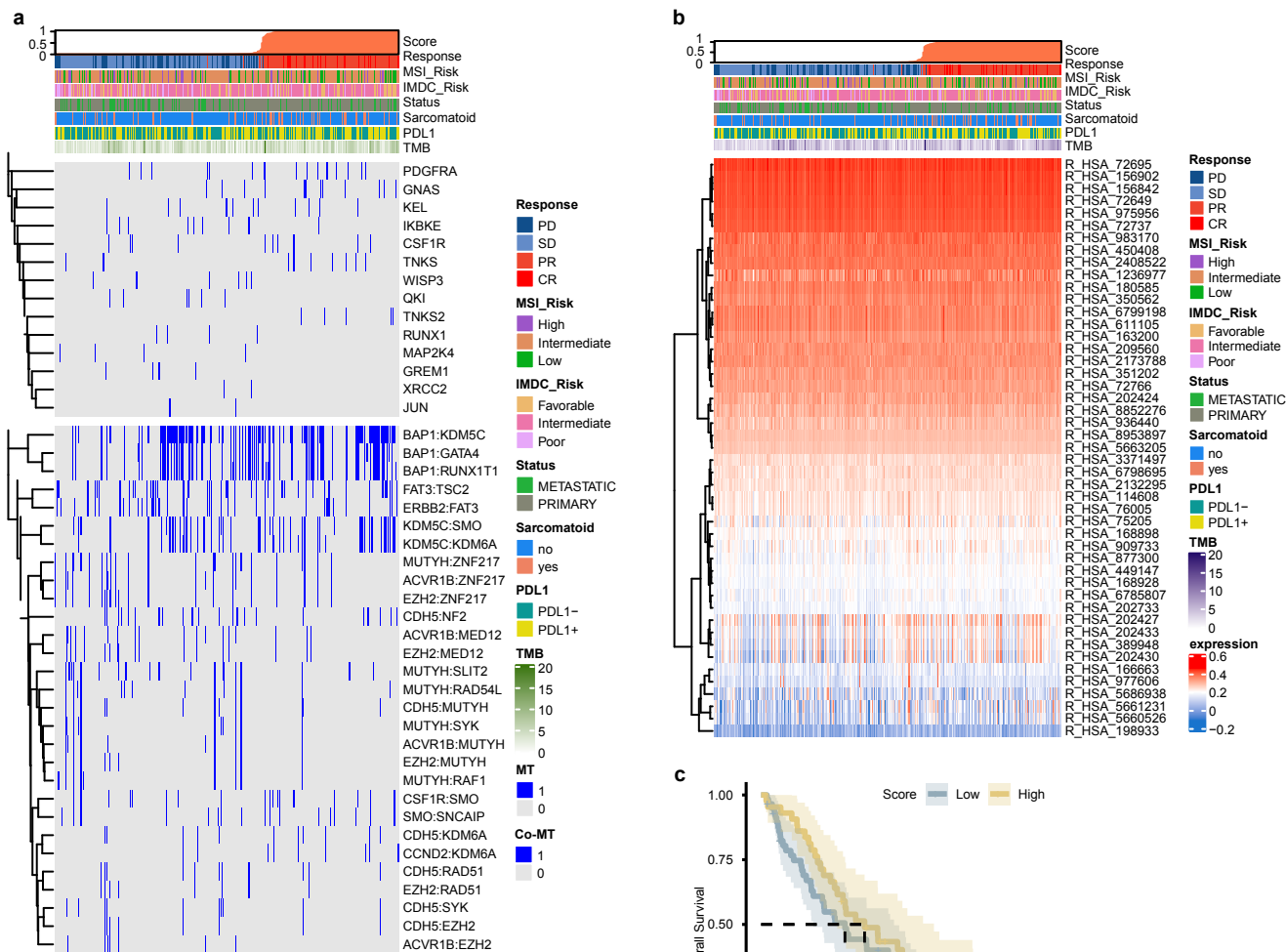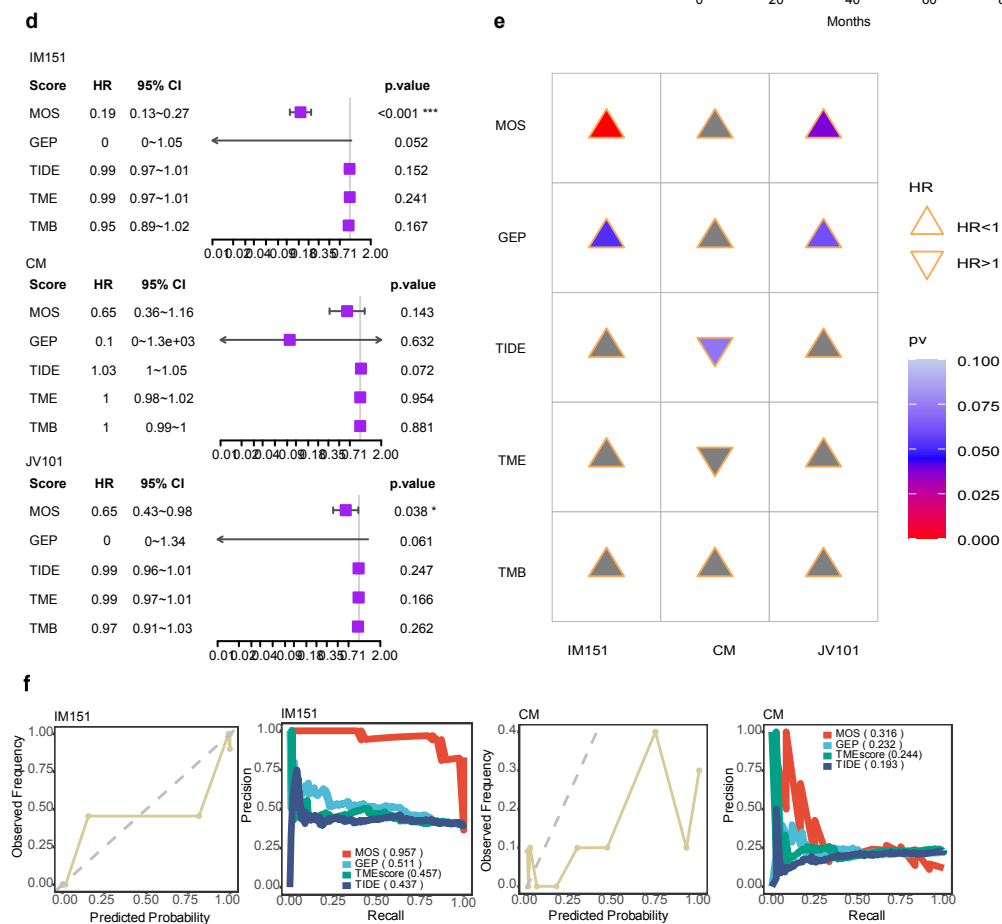

Supplement: Supplementary file 1 [file Presentation1.zip › Supplementary material presentation/Fig S6.pdf]

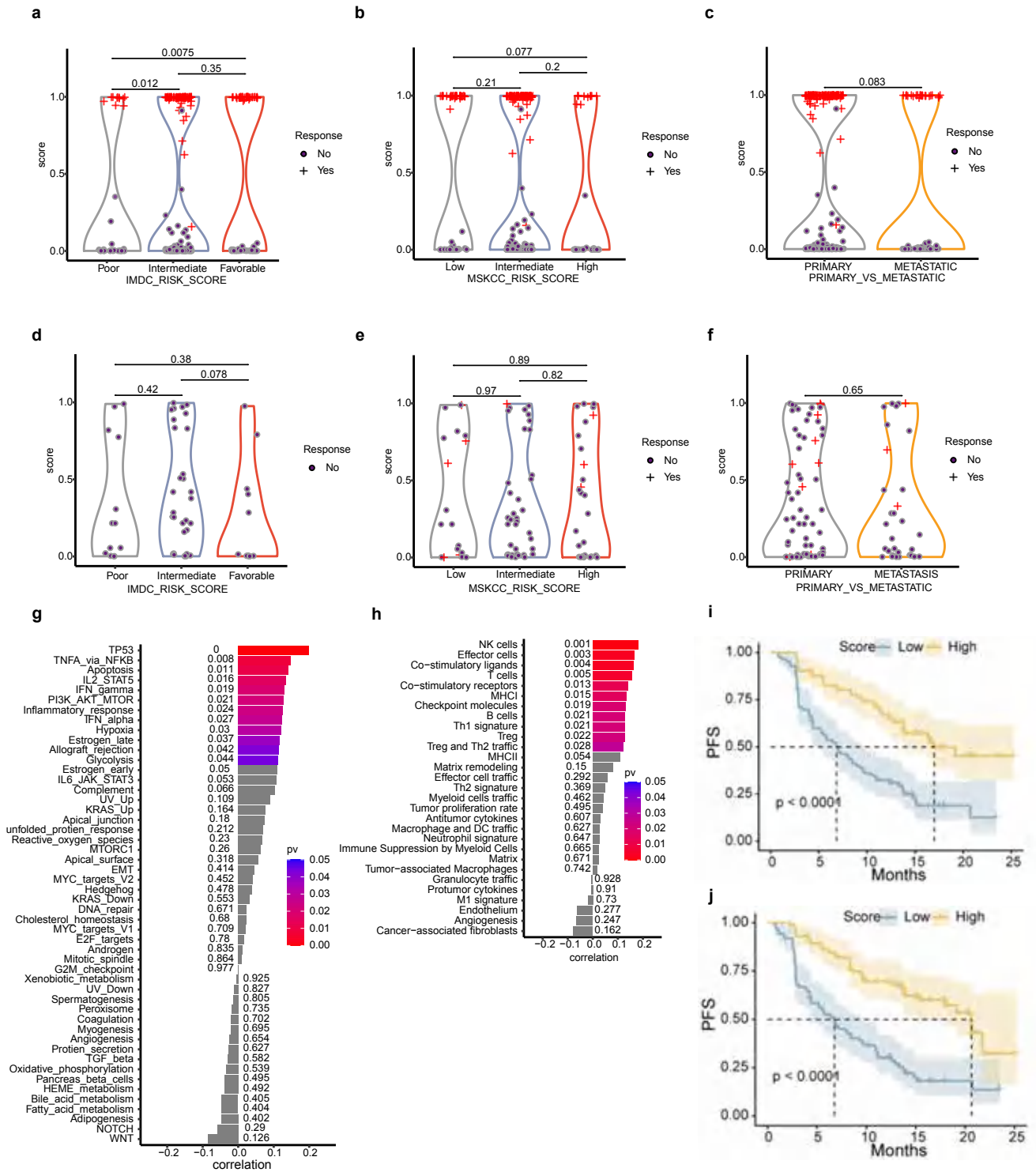

Supplement: Supplementary file 1 [file Presentation1.zip › Supplementary material presentation/Fig S7.pdf]

**a**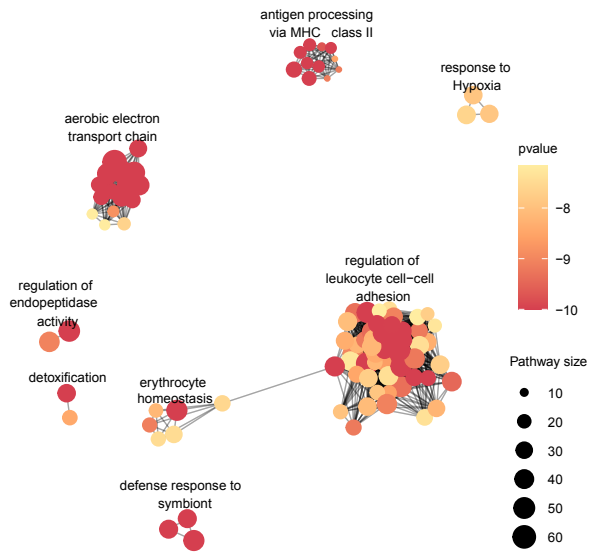**b**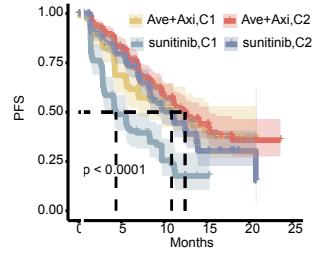**c**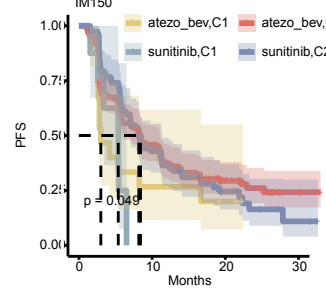**d**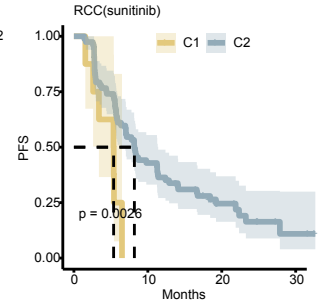**d**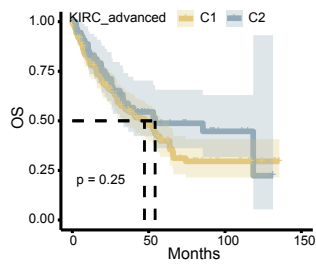**e**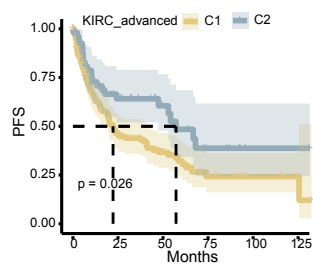**f**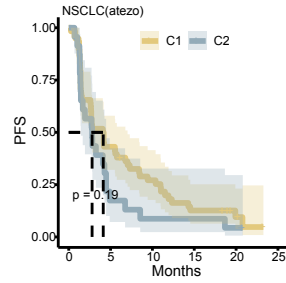**g**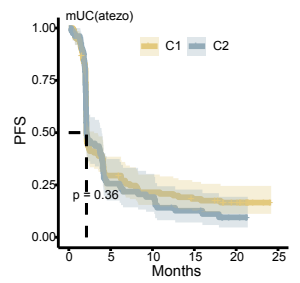

Supplement: Supplementary file 1 [file Presentation1.zip › Supplementary material presentation/Fig S9.pdf]

**a**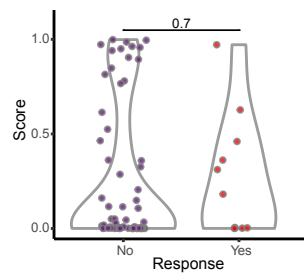**b**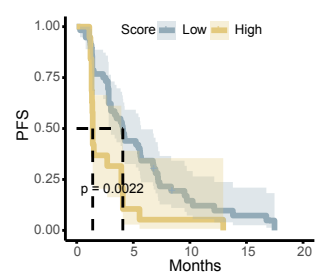**c**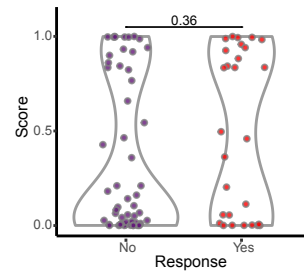**d**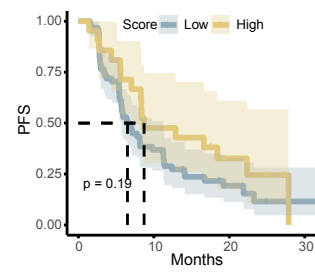

Supplement: Supplementary file 1 [file Presentation1.zip › Supplementary material presentation/Fig S8.pdf]
